# Physical–Chemical Properties of Biogenic Selenium Nanostructures Produced by *Stenotrophomonas maltophilia* SeITE02 and *Ochrobactrum* sp. MPV1

**DOI:** 10.3389/fmicb.2018.03178

**Published:** 2018-12-19

**Authors:** Elena Piacenza, Alessandro Presentato, Emmanuele Ambrosi, Adolfo Speghini, Raymond J. Turner, Giovanni Vallini, Silvia Lampis

**Affiliations:** ^1^Environmental Microbiology and Microbial Biotechnology Laboratory, Department of Biotechnology, University of Verona, Verona, Italy; ^2^Department of Biological Sciences, University of Calgary, Calgary, AB, Canada; ^3^Department of Molecular Sciences and Nanosystems, Ca’Foscari University, Venezia, Italy; ^4^Nanomaterials Research Group, Department of Biotechnology, University of Verona and INSTM, Verona, Italy

**Keywords:** biogenic nanomaterials, selenium, selenite, nanoparticles, nanorods, *Stenotrophomonas maltophilia* SeITE02, *Ochrobactrum* sp. MPV1, photoluminescence

## Abstract

*Stenotrophomonas maltophilia* SeITE02 and *Ochrobactrum* sp. MPV1 were isolated from the rhizosphere soil of the selenium-hyperaccumulator legume *Astragalus bisulcatus* and waste material from a dumping site for roasted pyrites, respectively. Here, these bacterial strains were studied as cell factories to generate selenium-nanostructures (SeNS) under metabolically controlled growth conditions. Thus, a defined medium (DM) containing either glucose or pyruvate as carbon and energy source along with selenite (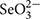) was tested to evaluate bacterial growth, oxyanion bioconversion and changes occurring in SeNS features with respect to those generated by these strains grown on rich media. Transmission electron microscopy (TEM) images show extra- or intra-cellular emergence of SeNS in SeITE02 or MPV1 respectively, revealing the presence of two distinct biological routes of SeNS biogenesis. Indeed, the stress exerted by 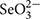 upon SeITE02 cells triggered the production of membrane vesicles (MVs), which surrounded Se-nanoparticles (SeNPs_SeITE02-G_e_ and SeNPs_SeITE02-P_e_ with average diameter of 179 ± 56 and 208 ± 60 nm, respectively), as highlighted by TEM and scanning electron microscopy (SEM), strongly suggesting that MVs might play a crucial role in the excreting mechanism of the SeNPs in the extracellular environment. On the other hand, MPV1 strain biosynthesized intracellular inclusions likely containing hydrophobic storage compounds and SeNPs (123 ± 32 nm) under pyruvate conditioning, while the growth on glucose as the only source of carbon and energy led to the production of a mixed population of intracellular SeNPs (118 ± 36 nm) and nanorods (SeNRs; average length of 324 ± 89). SEM, fluorescence spectroscopy, and confocal laser scanning microscopy (CLSM) revealed that the biogenic SeNS were enclosed in an organic material containing proteins and amphiphilic molecules, possibly responsible for the high thermodynamic stability of these nanomaterials. Finally, the biogenic SeNS extracts were photoluminescent upon excitation ranging from 380 to 530 nm, whose degree of fluorescence emission (λ_em_ = 416–640 nm) was comparable to that from chemically synthesized SeNPs with L-cysteine (L-cys SeNPs). This study offers novel insights into the formation, localization, and release of biogenic SeNS generated by two different Gram-negative bacterial strains under aerobic and metabolically controlled growth conditions. The work strengthens the possibility of using these bacterial isolates as *eco-friendly* biocatalysts to produce high quality SeNS targeted to possible biomedical applications and other biotechnological purposes.

## Introduction

The chalcogen selenium (Se) is an element that possesses intriguing physical–chemical properties due to its ability to behave as both metal and non-metal, therefore defined as metalloid (Haynes, 2014). This bivalent nature of Se relies on its existence in different allotropic forms, namely: amorphous Se (*a*-Se) that exists either as brick-red dust or black vitreous shape, which is generally obtained by means of reduction reactions, and crystalline Se, such as the gray hexagonal form (*h*-Se) and the less stable red monoclinic state (*m*-Se) (Habashi, 2013). Thanks to these structural peculiarities, Se has a very broad spectrum of applications in chemical, electrical and electronic industries, as it is characterized by a high photo- and thermal-conductivity, as well as electrocatalytic and photovoltaic activities (Chaudhary et al., 2016). Furthermore, Se is characterized by biological relevant properties, being an essential micronutrient contributing to the proper metabolic functioning in many living organisms (Reyes et al., 2006). Indeed, it is fundamental for the catalytic activity of glutathione (GSH) peroxidases (Mehdi et al., 2013), the detoxification of harmful compounds by GSH *S*-transferase enzyme (El-Bayoumy and Sinha, 2004), the prevention of DNA oxidation, and it is efficacious as both anticancer (Tinggi, 2008) and nutritional complement drug (Holubova et al., 2006), therefore resulting suitable for biomedical and pharmaceutical purposes (Chaudhary et al., 2016).

The significance and technological potential of Se is greatly improved when this element is scaled down to the nanorange (1–100 nm), emphasizing its peculiar physical–chemical properties (i.e., catalytic, mechanical, electrical, and opt-magnetic properties) (Appenzeller, 1991; Yuwen and Wang, 2013), due to the high surface-to-volume ratio, large surface energy and high spatial confinement of nanomaterials (Cao, 2004). In this respect, advances in the nanotechnology field led to the development of facile methods to produce Se-based nanomaterials (Brus, 1998), which found applications in diagnostics, electronic devices, catalysis, production of fuel cells, and environmental remediation (Chaudhary et al., 2016). However, most of the synthetic procedures considered so far require the use of toxic and harsh chemicals, resulting in the formation of hazardous waste, negatively impacting both human health and environment (Zhang et al., 2006). Moreover, these chemical practices involve the use of expensive equipments and chemical substances strongly affecting the production costs (Piacenza et al., 2018b); hence, we are now witnessing a growing demand for new, safe, economic and *eco-friendly* strategies to generate valuable Se-nanomaterials (Ankamwar et al., 2005).

The alternative *green* frontier in Se-nanostructures synthesis is represented by the exploitation of biological systems (plants, bacteria, fungi, yeasts, and algae) to bioconvert toxic Se-oxyanions (i.e., Selenate – 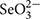 – and Selenite – 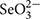) into their less toxic elemental forms (Se^0^) producing nanostructures (Zannoni et al., 2008). Particularly, bacteria are among the most explored organisms for Se-nanomaterials biosynthesis, due to their ability to colonize, adapt to and persist in adverse environmental niches (Li et al., 2011). In this context, both Gram-positive and -negative bacteria displayed their proficiency to biosynthesize intra-, extra-cellular and membrane bound Se-nanostructures (SeNS) as one of the mechanisms of oxyanion detoxification (Kessi et al., 1999; Wadhwani et al., 2016). The majority of Se-oxyanion bioconverting bacteria investigated so far, were able to produce Se-nanoparticles (SeNPs) ranging in size between 50 and 500 nm (Shirsat et al., 2015), while other biogenic Se nanomorphology (e.g., Se-nanorods – SeNRs) were observed in the case of few microorganisms (i.e., *Pseudomonas alcaliphila*, *Streptomyces bikiniensis* strain Ess_amA-1, *Bacillus subtilis*, *Ralstonia eutropha*, *Rhodococcus aetherivorans* sp. BCP1) (Wang et al., 2010; Zhang et al., 2011; Ahmad et al., 2015; Srivastava and Mukhopadhyay, 2015; Presentato et al., 2018a). The so-called biogenic SeNS showed to be efficacious as both antimicrobial agents capable of inhibiting the proliferation of pathogenic microorganisms growing as planktonic cells and/or forming biofilms (Oves et al., 2013; Zonaro et al., 2015; Cremonini et al., 2016; Piacenza et al., 2017), and anticancer agents against human tumor cell lines (Ahmad et al., 2015), therefore emphasizing the biotechnological relevance of these NS.

Another undisputed property of quantum confined metalloids is the optical one, and from a chemical perspective, Se-based nanomaterials have proven suitability as optoelectronic devices (Chaudhary et al., 2016). However, this characteristic for the biogenic Se counterparts have yet to be explored, representing still to date an important gap to be filled to explore their possible use in different fields (e.g., electronics and biomedicine, as advanced and innovative cell imaging tools).

In the present study, *Stenotrophomonas maltophilia* SeITE02 and *Ochrobactrum* sp. MPV1, previously isolated from different environmental matrices (Di Gregorio et al., 2005; Lampis et al., 2015), were evaluated for their ability to handle 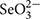 toxicity under metabolically controlled growth conditions. Our aim was to expand the knowledge about the bacterial physiology of these two strains as compared to earlier studies performed under optimum growth conditions in rich medium (Lampis et al., 2017; Zonaro et al., 2017). Here we observe SeITE02 and MPV1 strains elicited different bioprocess mechanisms to cope with 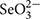 toxicity under metabolically controlled growth conditions, which in turn determined the synthesis of Se-nanomaterials featured by different morphologies. The biogenic extracts containing SeNS were then characterized from a physical–chemical perspective to elucidate macromolecular composition and thermodynamic stability of the biogenic nanostructures, as well as to shed light on their photoluminescence (PL) properties, as proof of concept of their suitability as novel optical imaging tool for biotechnological purposes.

## Materials and Methods

### Bacterial Cultures and Growth Conditions

The environmental bacterial isolates *Stenotrophomonas maltophilia* SeITE02 and *Ochrobactrum* sp. MPV1 were routinely pre-cultured in Luria Bertani [hereafter named as LB, containing (g L^-1^): sodium chloride (10, Sigma-Aldrich^®^), tryptone (10, Oxoid^TM^), yeast extract (5, Oxoid^TM^); when needed LB medium was solidified by adding 15 g L^-1^ of Agar (Oxoid^TM^)] medium for 16-h at 27°C with shaking (200 rpm). SeITE02 and MPV1 strains were then inoculated (1% v/v) in 250 mL Erlenmeyer flasks containing 50 mL of Defined Medium (hereafter named as DM; Frassinetti et al., 1998) amended with glucose and/or sodium pyruvate (0.5% v/v, Sigma-Aldrich^®^) as the only source of carbon and energy, with or without 0.5 mM sodium selenite (Na_2_SeO_3_, Fluka^®^). To estimate the bacterial growth, every 24-h (up to 120-h) an aliquot of cells (100 μL) was serially diluted and plated onto LB agar recovery plates, in order to count the colony forming unit (CFU mL^-1^), which is reported in logarithm scale with standard deviations (*n* = 3), for each experimental condition tested.

### Biotic Conversion of 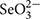


The 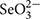 concentration was evaluated over the incubation time as published elsewhere (Kessi et al., 1999), by sampling both SeITE02 and MPV1 culture broths every 24-h of growth. The residual oxyanion concentrations are reported as average values (*n* = 3) with standard deviations.

### Preparation and Recovery of the Biogenic SeNS Extracts and Chemical SeNPs

The biogenic SeNS extracts were prepared by means of two different methods, depending on the type strain considered. Since SeITE02 strain produced SeNPs extracellularly, SeITE02 biomass was centrifuged (3000 *g* for 20 min) after 120-h of growth to recover the cell-free spent medium, which was filtered using 0.20 μm Filtropur (Sarstedt). After this step, the filtered solution containing SeNPs was centrifuged (20,000 *g* for 30 min) to collect the biogenic nanomaterial, which was then resuspended in sterile distilled water. On the other hand, since MPV1 strain produced Se-nanomaterial intracellularly, the cells were (i) collected through centrifugation (3000 *g* for 20 min), (ii) resuspended in 10 mL of 1.5 mM Tris-HCl (Sigma-Aldrich^®^) buffer (pH 7), and (iii) disrupted by ultrasonication (UP50H hielscher) at 50 W for 5 min, performing 30 s of burst interspersed by 30 s of pause on ice. Finally, the cellular debris was removed by centrifugation (3000 *g* for 20 min) and the supernatant containing SeNS was treated as described above, in order to collect the biogenic nanomaterial.

Since the biogenic SeNS extracts were generated utilizing two different bacterial isolates grown under two different conditions, for clarity, in the Supplementary Table S1 is reported a summary of the bacterial growth conditions tested to generate the biogenic SeNS extracts, as well as their acronym.

L-Cysteine SeNPs (L-cys SeNPs) were prepared as described by Li et al. (2010). Briefly, L-cysteine (50 mM) and sodium selenite (100 mM) solutions were mixed in a ratio 4:1 at room temperature, in order to obtain a population of SeNPs ranging in size between 150 and 200 nm, similarly to those biogenically synthesized.

### Electron Microscopy (EM) and Energy-Dispersive X-ray Spectroscopy (EDX) Analyses

SeITE02 and MPV1 cells grown in the presence of 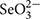 were imaged by collecting (every 24-h) an aliquot (500 μL) of cells that was washed three times (10 min each) with distilled water to remove the excess of salts deriving from the culture broth. Then, 5 μL of cells resuspended in distilled water were deposited onto carbon coated copper grids (CF300-CU, Electron Microscopy Sciences), which were then air-dried prior their visualization by means of a Philips CM100 transmission electron microscope (TEM).

The morphological and elemental analyses of SeNS extracts were carried out using a Zeiss Sigma VP field emission scanning electron microscope (FESEM) and a Bruker XFlash^®^ 4 detector, respectively. In this respect, the biogenic SeNS extracts were washed with ethanol and hexane/water solution (Sigma-Aldrich^®^) to remove excesses of organic contaminants, then 2 μL of each extract deposited onto crystal silicon wafers (type N/Phos, size 100 mm, University Wafer) and dried at room temperature, prior the imaging. To establish the actual average diameter and/or length of SeNS, 100 randomly chosen nanostructures were measured using ImageJ software.

### Confocal Laser Scanning Microscopy (CLSM) and Photoluminescence (PL) Analyses

The biogenic SeNS extracts were labeled by using the lipophilic tracer DiOC_18_(3) as published elsewhere (Presentato et al., 2018b). Subsequently, 20 μL of the labeled samples were deposited on microscopy glass slides and air dried, being then imaged using a LEICA model DM IRE2 confocal laser scanning microscope (CLSM) exploiting a 488-nm laser source.

The fluorescence emission and excitation spectra of the labeled samples as well as the PL properties of L-cys SeNPs and the unlabeled biogenic SeNS extracts were collected using a Nanolog/Fluorolog-3-2iHR320 modular spectrofluorimeter.

## Results

### SeITE02 Growth, 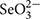 Bioconversion, and Biosynthesis of SeNS

**FIGURE 1:**
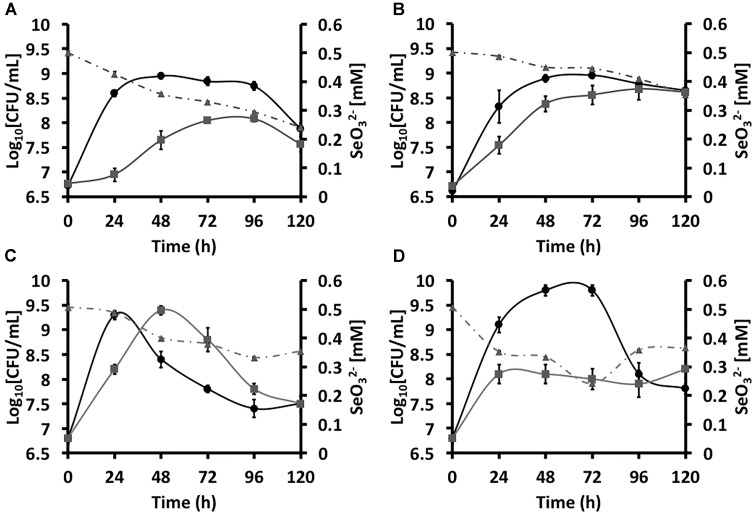
Growtheee4 curvesddd5 of *Stenotrophomonas maltophilia* SeITE02 **(A,B)** and *Ochrobactrum* sp. MPV1 **(C,D)** in DM amended with glucose and/or pyruvate (black curves), and with the addition of 0.5 mM 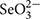 (gray curves). The gray dashed curves represent the biotic conversion of 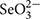 over the timeframe considered.

The addition of 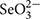 to glucose growing cultures of SeITE02 led to a 24-h lag phase, which was absent in the case of those not amended with the oxyanions (Figure 1A). On the other hand, SeITE02 growth profile on pyruvate did not differ regardless 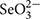 exposure (Figure 1B).

Different extents of 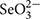 consumption were observed as function of the carbon source supplied to the growth medium (Figures 1A,B), partially occurring the bioconversion of 0.5 mM of oxyanions within 120-h of growth. Indeed, SeITE02 glucose-grown cells consumed higher amounts of 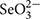 (0.26 mM) as compared to those grown on pyruvate, where the extent of 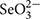 bioconversion was 0.15 mM.

**FIGURE 2:**
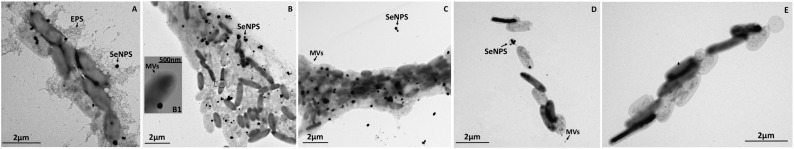
Time course imaging by transmission electron microscopy (TEM) of *Stenotrophomonas maltophilia* SeITE02 strain grown in DM amended with glucose (0.5% v/v) and 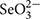 (0.5 mM) for 24-h **(A)**, 48-h **(B)**, 72-h **(C)**, 96-h **(D),** and 120-h **(E)**. The inset **(B1)** shows an enlargement of a cell featured by membrane vesicles (MVs), which is indicated by black arrow, as well as other features such as: extra polymeric substance (EPS) and selenium nanoparticles (SeNPs).

**FIGURE 3:**
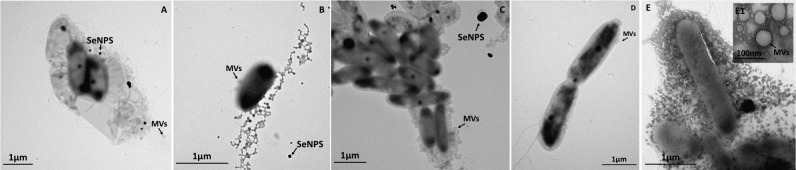
Time course imaging by TEM of *Stenotrophomonas maltophilia* SeITE02 strain grown in DM amended with pyruvate (0.5% v/v) and 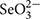 (0.5 mM) for 24-h **(A)**, 48-h **(B)**, 72-h **(C)**, 96-h **(D)**, and 120-h **(E)**. The inlet **(E1)** shows an enlargement of microbial vesicles (MVs), which is indicated by black arrow, as well as SeNPs.

Regardless the carbon source utilized to support SeITE02 growth, this strain showed the capability of producing SeNPs consequently to 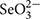 bioconversion (Figures 2, 3). Although some intracellular SeNPs were detected in SeITE02 cells after 24-h of growth upon exploitation of both carbon sources supplied (Figures 2A, 3A), these nanostructures were mostly released into the extracellular environment over the timeframe considered (from 24 to 120-h). Further, biogenic SeNPs were surrounded by membrane vesicles (MVs; Figures 2B,B1, 3A,B), which were produced by SeITE02 cells along with extracellular polymeric substance (EPS; Figure 2A) as stress response to 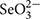 during the earliest stages of growth (24 and 48-h) on either glucose or pyruvate. This phenomenon appeared to be exacerbated at 120-h of growth upon pyruvate conditioning of the bacterial cells (Figure 3E).

### MPV1 Growth, 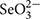 Bioconversion, and Biosynthesis of SeNS

MPV1 strain was differently influenced with respect to SeITE02 by the presence of 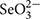 added to the culture broth amended with either glucose or pyruvate as the only source of carbon and energy (Figures 1C,D). Indeed, MPV1-glucose grown culture under 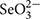 pressure displayed a 24-h time-shifted growth as compared to that not exposed to the oxyanions, eventually reaching an equal biomass yield; further, both batch cultures showed a linear decrease of the CFU mL^-1^ over the incubation time (Figure 1C). In the case of MPV1 cells grew on pyruvate, the bacterial growth was featured by a delayed cell death, as observed in the case of MPV1-glucose grown cells, while under 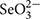 stress the culture reached a plateau after 24-h of incubation and a low biomass yield over the experimental timeframe (Figure 1D).

Similarly to SeITE02, MPV1 cells partially consumed 0.5 mM 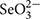 within the incubation time considered (Figures 1C,D), bioconverting comparable amount of oxyanions i.e., 0.20 and 0.18 mM in the case of cells grew either on glucose or pyruvate, respectively.

**FIGURE 4:**
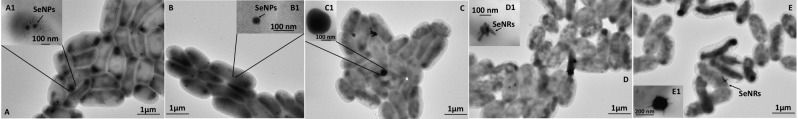
Time course imaging by TEM of *Ochrobactrum* sp. MPV1 strain grown in DM amended with glucose (0.5% v/v) and 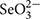 (0.5 mM) for 24-h **(A)**, 48-h **(B)**, 72-h **(C)**, 96-h **(D)**, and 120-h **(E)**. The inlets **(A1–E1)** show enlargements of intracellular SeNPs and nanorods (SeNRs), which are indicated by black arrows.

**FIGURE 5:**
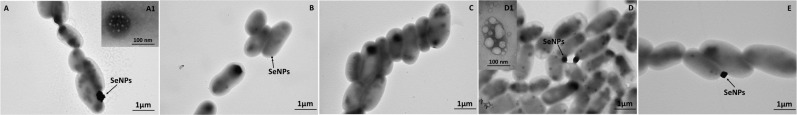
Time course imaging by TEM of *Ochrobactrum* sp. MPV1 strain grown in DM amended with pyruvate (0.5% v/v) and 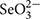 (0.5 mM) for 24-h **(A)**, 48-h **(B)**, 72-h **(C)**, 96-h **(D)**, and 120-h **(E)**. The inlets **(A1,D1)** show intracellular inclusions. SeNPs are indicated by black arrows.

As a result of 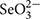 bioconversion, MPV1 strain was capable of producing selenium nanomaterials with different morphologies, depending on the carbon source exploited to sustain bacterial growth (Figures 4, 5). Indeed, while MPV1-pyruvate grown cells generated SeNPs (Figure 5), a mixed population of NPs and NRs was detected when MPV1 grew on glucose as sole carbon and energy source, most likely due to NPs dissolution, as shown in Figures 4 (**D,E** and inlets **D1,E1**). Overall, MPV1 biosynthesized intracellular SeNS (Figures 4, 5), which were either homogenously distributed within the cells or located mainly on the outer cellular surfaces (Figures 4, 5). Moreover, intracellular organic accumulations were produced by this strain from 24-h onwards (Figures 5A1 and inlet **D1**) when grown in the presence of pyruvate and 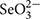. These cellular inclusions were sphere-shaped and electron-transparent (Figures 5 inlets **A1,D1**) as those observed by Alvarez et al. (1996) in the case of the hydrocarbon-degrading *Rhodococcus opacus* PD630 strain (Alvarez et al., 1996). None of these intracellular structures were observed in MPV1 cells under glucose growth condition (Figure 4).

Overall, the data reported so far suggest different strategies adopted by SeITE02 and MPV1 strains to adapt, interface and bioprocess 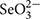, as well as the carbon source supplied.

### Bio-chemical and -Physical Features of SeNS Extracts

**FIGURE 6:**
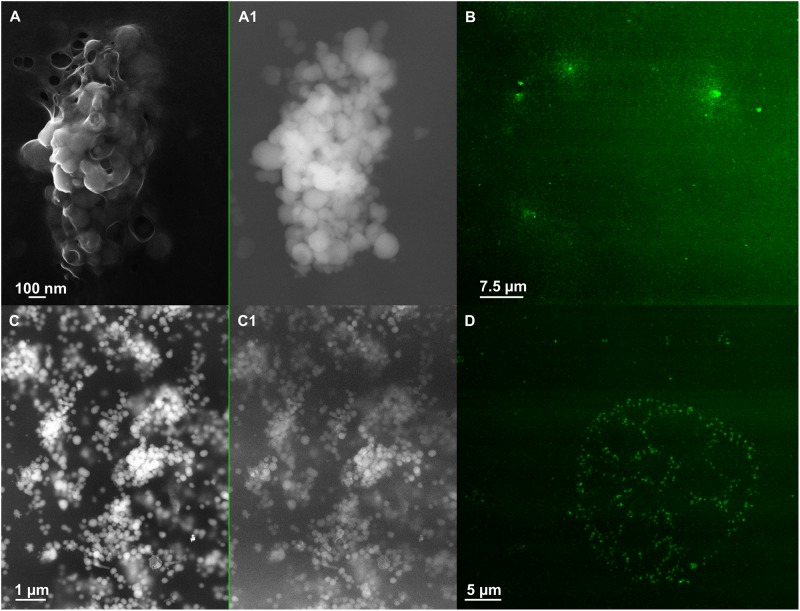
Scanning electron microscopy (SEM) of SeNPs_SeITE02-G_e_ [**A** (In Lens detector), **A1** (detector for back scattered electron)] and SeNPs_SeITE02-P_e_ [**C** (In Lens detector), **C1** (detector for back scattered electron)]. Confocal laser scanning microscopy (CLSM) of SeNPs_SeITE02-G_e_ **(B)** and SeNPs_SeITE02-P_e_ **(D)** labeled with the lipophilic tracer DiOC_18_(3).

**FIGURE 7:**
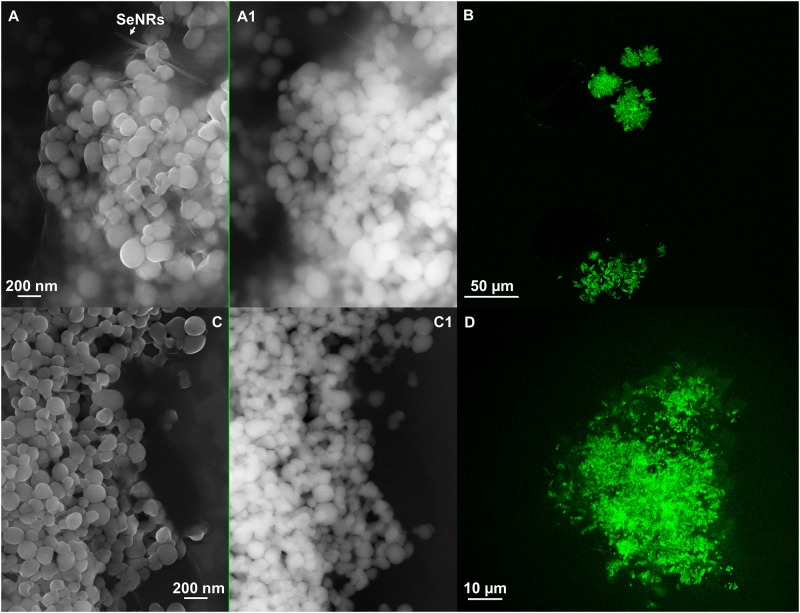
Scanning electron microscopy of SeNS_MPV 1-G_e_ [**A** (In Lens detector), **A1** (detector for back scattered electron)] and SeNPs_MPV 1-P_e_ [**C** (In Lens detector), **C1** (detector for back scattered electron)]. Confocal laser scanning microscopy (CLSM) of SeNS_MPV 1-G_e_ **(B)** and SeNPs_MPV 1-P_e_ **(D)** labeled with the lipophilic tracer DiOC_18_(3).

The biogenic SeNS extracts were analyzed by using scanning electron microscopy (SEM), EDX, fluorescence spectroscopy and CLSM, in order to shed light on their morphological and physical–chemical features. In this regard, the average diameter and length of SeNPs and NRs of the extracts SeNPs_SeITE02-G_e_, SeNPs_SeITE02-P_e_, SeNPs_MPV 1-P_e_, and SeNS_MPV 1-G_e_ are reported in Table 1. A morphological feature shared among the biogenic SeNS extracts was the presence of a material composed by light elements that enclosed the SeNS (Figures 6A,C, 7A,C), preventing their aggregation even upon deposition onto silicon (Si) wafers. To support this hypothesis, the elemental composition of SeNS extracts was evaluated by EDX spectroscopy. Since the extracts containing the biogenic nanostructures were dried onto silicon wafers, the Si signal (K_α_ = 1.739 KeV) was obtained for all analyzed samples (Supplementary Figure S2), while the presence of sodium (Na; K_α_ = 1.041 KeV; Supplementary Figure S2a) most likely depended on the extraction protocol used, which involved sodium salts. EDX spectra showed the presence of two Se peaks (K_α_ = 11.207 KeV and L_α_ = 1.379 KeV), one signal for carbon (C; K_α_ = 0.277 KeV) and oxygen (O; K_α_ = 0.525 KeV) (Supplementary Figure S2) in all the biogenic nanomaterial extracts, while sulfur (S; K_α_ = 2.307 KeV) and phosphorous (P; K_α_ = 2.013 KeV) signals were detected only in the case of those extracts deriving from SeITE02 cell-free spent media (Supplementary Figures S2a,b). Thus, the elemental composition analysis of the biogenic SeNS extracts indicated that the material enclosing the NS was of organic nature.

**Table 1:** Average diameter and length (nm) of SeNS generated by SeITE02 and MPV1 cells grown for 120-h in the presence of 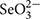 and either glucose or pyruvate as the only source of carbon and energy.

	Average diameter/length (nm)	
Sample	SeNPs	SeNRs
SeNPs_SeITE02-G_e_	179 ± 56	ND^a^
SeNPs_SeITE02-P_e_	208 ± 60	ND
SeNS_MPV 1-G_e_	118 ± 36	324 ± 89
SeNS_MPV 1-P_e_	123 ± 32	ND

^a^*None detected*.

To better elucidate the biomolecular composition of the organic material surrounding the biogenic SeNS, fluorescence spectroscopy was performed. The presence of proteins was assessed exciting the biogenic SeNS extracts at 280 nm, resulting in a sharp fluorescence emission peak at 325 nm typical of the amino acid tryptophan in proteins (Supplementary Figure S3a; Lakowicz, 1999), while L-cys SeNPs, which were utilized as chemical comparison, showed only the water Raman peak contribution (308 nm; Supplementary Figure S3b) (Bartlett et al., 1998).

**FIGURE 8:**
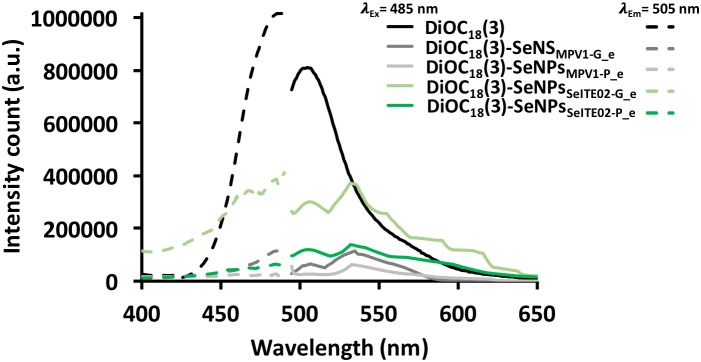
Fluorescence emission and excitation spectra of DiOC_18_(3) labeled biogenic SeNS extracts.

The amphiphilic nature of the organic layer surrounding the biogenic nanostructures was assessed by labeling the extracts with the lipophilic tracer DiOC_18_(3). Particularly, the specificity of this dye relies on its capability to emit fluorescence in an apolar solvent or when it is bound to hydrophobic moieties, being otherwise the free tracer molecules quenched in aqueous polar environment (Huaglang, 2002; Yefimova et al., 2008). The interaction between the lipophilic tracer and the amphiphilic moieties present in the samples, as well as the potential formation of organized structures resulting from this interaction, was assessed by performing CLSM. The labeled extract SeNPs_SeITE02-G_e_ showed diffuse green fluorescence (Figure 6B), suggesting a uniform distribution of the organic layer, while regular ring-like green fluorescent structures were observed in the case of DiOC_18_(3)-SeNPs_SeITE02-P_e_ (Figure 6D), therefore indicating a peculiar organization of the amphiphilic molecules around SeNPs. On the other hand, the fluorescent signals of the labeled SeNS_MPV 1-G_e_ and SeNPs_MPV 1-P_e_ extracts resulted to be localized around the nanomaterial content (Figures 7B,D). Particularly, DiOC_18_(3)-SeNS_MPV 1-G_e_ displayed green fluorescent needle-like structures (Figure 7B), which resembled the SeNRs previously detected through EM (Figures 7A,A1 and Supplementary Figure S1). Furthermore, since DiOC_18_(3) dissolved in ethanol showed a fluorescence emission peak at 505 nm, the fluorescence signal of the DiOC_18_(3) labeled samples detected at 507 nm (Figure 8) suggested that an interaction between the lipophilic tracer and the amphiphilic molecules of the biogenic extracts occurred. These results were corroborated by the fluorescence excitation spectra that displayed the main peak centered at 485 nm (Figure 8 and Supplementary Figure S4), which corresponds to the excitation wavelength of DiOC_18_(3). A second emitting species contributing to a broad fluorescent emission peak (λ_em_ = 530 nm) was detected (Figure 8), which might be due to intrinsic photoluminescence (PL) properties of the biogenic selenium nanomaterials, discussed below.

### Photoluminescence (PL) Properties of Biogenic SeNS Extracts

**FIGURE 9:**
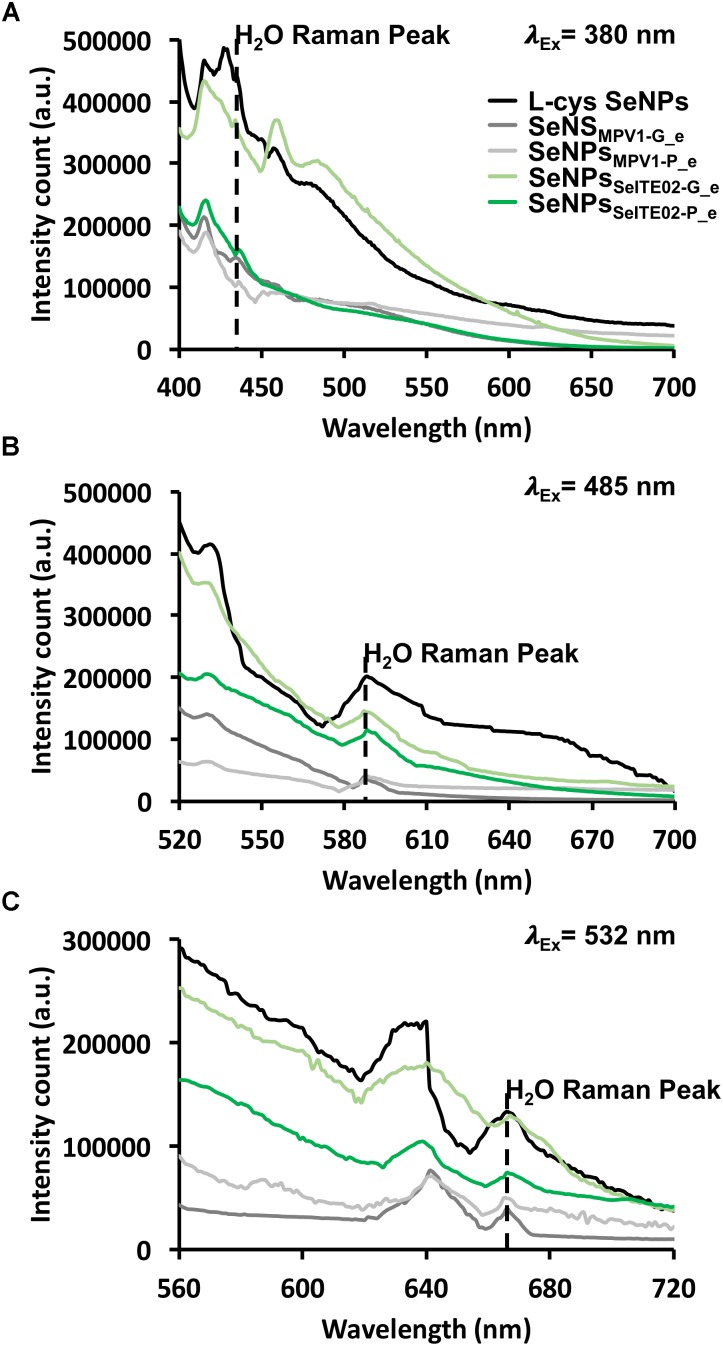
Photoluminescence emission spectra of the unlabeled chemically and biogenically synthesized SeNS at exitation wavelengths of 380 **(A)**, 485 **(B)**, and 532 nm **(C)**. The dashed black line indicates the water Raman peak.

To investigate the intrinsic PL properties of the biogenic SeNS extracts, fluorescence spectroscopy was performed on the unlabeled samples and chemically synthesized SeNPs (L-cys SeNPs; Li et al., 2010) to evaluate the photoluminescent behavior of Se at the nanoscale (Figure 9). As a result, L-cys SeNPs showed PL emission maxima at 416 and 428 nm upon excitation at 380 nm, while the highest PL signal for those of biogenic synthesis was centered at 416 nm (Figure 9A), which is in line with the PL data reported by Khalid et al. (2016) and Qian et al. (2017). Moreover, as the excitation wavelength increased (i.e., λ_exc_ = 485 or 532 nm) a red-shift of the PL emission maxima (λ_em_ = 530 or 640 nm) was observed (Figures 9B,C). Thus, the PL emission peak at 530 nm detected upon excitation at 485 nm (Figure 9B) indicated that the fluorescent signal previously observed for the DiOC_18_(3) labeled biogenic SeNS extracts (Figure 8) was due to the intrinsic PL properties of Se in nanosized form.

The SeNS PL dependency on the excitation wavelength was assessed by systematically moving 20 nm steps the λ_exc_ (from 380 to 589 nm; Supplementary Table S2). In this regard, the PL peak position maxima red-shifted from 416 or 428 to 680 nm within the range of the λ_exc_ tested, showing a linear relationship between the variation of the PL emission and the excitation wavelength (Supplementary Figure S5 and Table S2). The PL excitation (PLE) spectra confirmed the PL dependency on the excitation wavelength, resulting in the detection of PLE maxima at 485 or 532 nm when either 530 or 640 nm were set as emission wavelengths (Supplementary Figures S6b,c). Additionally, a progressive increase (ca. 20 nm interval) of the λ_em_ (from 416 to 640 nm) yielded PLE peaks whose position red-shifted from 380 to 532 nm (Supplementary Table S3), being λ_em_ and λ_exc_ linearly correlated (Supplementary Figure S7), thus supporting the PL results described above.

As further aid to appreciate the results here, a list of the key observations highlighting both similarities and differences occurring between SeITE02 and MPV1 is supplied as Table 2.

**Table 2:** Synoptic report that at glance supply evidences regarding the microbial physiology of SeITE02 and MPV1 strains, as well as physical–chemical and biological features of selenium nanomaterials.

*Stenotrophomonas maltophilia* SeITE02		*Ochrobactrum* sp. MPV1	
**Growth of the environmental isolates under metabolically controlled conditions**			

• Glucose and pyruvate are the sources of carbon and energy exploited by bacterial cells to grow either in the presence or absence of 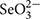 as selenium precursor;			
• Both strains can biotically convert 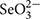 synthesizing selenium nanomaterials			

**Occurrence of selenium nanostructures**			

• Intracellular		• Extracellular and on the outer cellular surface	

**Physiology features and morphology of the biogenic nanomaterial as function of the carbon source**			

**Glucose**	**Pyruvate**	**Glucose**	**Pyruvate**

• Microbial vesicles (MVs);		• SeNPs;	• Intracellular inclusions;
		• SeNRs	• SeNPs
• Extracellular Polymeric Substance (EPS);			
• SeNPs			

**Elemental composition of the organic coatingof selenium nanostructures**			

• Carbon;		• Carbon;	
• Oxygen;		• Oxygen	
• Sulfur;			
• Phosphorous			

**Macromolecules detected by fluorescence spectroscopy**			

• Proteins (λ_em_ = 325 nm);			
• Amphiphilic molecules upon labeling with DiOC_18_(3) (λ_em_ = 507 nm)			

**Photoluminescence properties of selenium nanostructures**			

• Photoluminescence emission maxima (λ_em_) at 416 nm (λ_exc_ = 380 nm);			
• Red-shift of photoluminescence emission centered at 530 or 640 nm upon excitation of the biogenic nanomaterial at 485 or 532 nm, respectively;			
• Linear dependency of the photoluminescence emission on the excitation wavelength			

## Discussion

The environmental robustness and resilience of SeITE02 and MPV1 isolates against 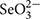 under optimized conditions of growth (Lampis et al., 2017; Zonaro et al., 2017) were earlier investigated. In the present study, these two strains were challenged under aerobic and metabolically controlled growth conditions, in order to shed light on possible biological routes leading to the synthesis and release of selenium nanomaterials.

The environmental isolates were capable of exploiting either glucose or pyruvate as the only source of carbon and energy, although MPV1 was strongly affected by the mere presence of the carbon sources supplied as compared to SeITE02 (Figure 1). Indeed, MPV1-glucose grown cells, and with a lesser extent those grown on pyruvate, had an evident death phase that started after 24 or 72-h of incubation, respectively (Figures 1C,D). A reasonable explanation of this phenomenon may rely on the metabolism of glucose in an oxic environment, as in a condition where glucose is in excess (ca. 0.5% w/v), the tricarboxylic acid cycle (TCA) is turned off, therefore determining pyruvate accumulation (Somerville et al., 2003). As a result, pyruvate produced during bacterial glycolysis can be in turn converted into acetic acid, which is secreted until the complete glucose depletion occurs (Patton et al., 2005). Thus, the uptake and catabolism of high amounts of acetic acid appears to be an important factor responsible for cell fate (life vs. death), as it can stimulate the murein hydrolase activity, therefore determining cell lysis (Rice et al., 2005; Rice and Bayles, 2008).

The growth profile of both strains was temporally shifted upon addition of 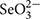 to the culture broth, and, even though any statistical difference was not observed between the CFU mL^-1^ at the latest time points considered, the biomass production was lower over the time course as compared to 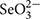 free cultures (Figure 1). Moreover, although these two bacterial strains were previously described for their ability to totally bioconvert 0.5 mM 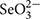 within 30 (MPV1) and 52-h (SeITE02) of growth in rich medium (Lampis et al., 2017; Zonaro et al., 2017), here, the initial oxyanion content was not entirely bioconverted by both bacterial strains in all the conditions tested (Figure 1). This aspect may be related to the toxicity exerted by 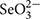 that relies on the generation of a strong cytoplasmic redox unbalance of the glutathione/glutaredoxin (GSH) and thioredoxin (RSH) pool (Carmel-Harel and Storz, 2000), which is considered to cause an increased production of intracellular reactive oxygen species (ROS; e.g., hydrogen peroxide), leading to cell death (Held and Biaglow, 1994). Thus, considering that in both SeITE02 and MPV1 strains 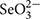 bioconversion mainly occurs by means of the cytoplasmic GSH pools exploiting either NADH or NADPH as electron donors (Lampis et al., 2017; Zonaro et al., 2017), the lower biomass yield produced under metabolically controlled growth, and, therefore, the decreased cellular GSH content, might explain the incomplete oxyanion consumption as compared to that observed under optimized conditions of growth.

The partial 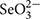 bioconversion performed by SeITE02 and MPV1 strains led to the production of either extracellular or intracellular Se-nanomaterials (Figures 2–5 and Supplementary Figure S1), respectively. Particularly, the biosynthesis of SeNPs by SeITE02 started within the cells during the earliest stage of incubation, while later on mainly extracellular SeNPs were detected, as already observed when this strain was grown under optimized conditions (Lampis et al., 2017), indicating that a sort of SeNPs expulsion mechanism must occur. Although the knowledge of the expulsion process of biogenic SeNPs is still in its infancy, the proposed mechanisms up to date are those involving (i) the active export through membrane-associated reductases (Losi and Frankenberger, 1997), (ii) the binding of SefA protein to SeNPs (Butler et al., 2012), (iii) cell lysis (Tomei et al., 1995) and (iv) the vesicular-mediated expulsion (Kessi et al., 1999). In line with the latter hypothesis, one of the routes most exploited by Gram-negative bacteria for the secretion of both insoluble and soluble material is the formation of MVs from the outer membrane, which normally occurs as bacterial stress response (McBroom and Kuehn, 2007; Kulp and Kuehn, 2010). In the present study, SeITE02 cells cultured under metabolically controlled conditions were characterized by a massive presence of MVs (Figures 2, 3), which were observed to surround the SeNPs (Figures 2D, 3C). These observations suggested that SeNPs expulsion might occur through a vesicular-mediated process, as proposed for *Rhodospirillum rubrum* (Kessi and Hanselmann, 2004) and *Saccharomyces cerevisiae* (Zhang et al., 2012; Pereira et al., 2018). Moreover, at the early stage of SeITE02 growth on glucose, EPS-like material was detected (Figure 2A), which represents a strategy adopted by bacteria to counteract the toxicity exerted by metal cations or metalloid oxyanions, due to the adsorptive potential of these biological exudates (Jixian et al., 2015; Zheng-Bo et al., 2015). On the other hand, the biosynthesis of a mixed population of SeNPs and NRs by MPV1 cells exploiting glucose as carbon and energy source (Figures 4D inlet D1,E, inlet E1 and Supplementary Figure S1) could be ascribed to the concomitantly production of surfactant-like molecules. The synthesis of these biologically active compounds was earlier reported for bacterial strains belonging to the *Ochrobactrum* genus growing under stress conditions (Kumar et al., 2014). Since the mere presence of glucose represented a condition of stress for MPV1 cells (Figure 1C), it is reasonable to think that the further stress derived upon 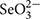 exposure might lead to the production of biosurfactants guiding the growth of SeNRs, which were not observed when MPV1 cells grew under optimized conditions (Zonaro et al., 2017). Indeed, the chemical production of NRs is generally achieved by the addition of surfactants in the reaction mixture, which, due to their amphiphilic nature, can adsorb onto the nanomaterial surface acting as both stabilizing agent and driving force stimulating the anisotropic growth of NRs in one direction (Evans and Wennerstrom, 1994; Eastoe and Tabor, 2014). This process appears to be facilitated for those elements, such as the metalloid Se, that can exist in amorphous or crystalline configuration, being the latter more thermodynamically stable and typical constituent of NRs (Jeong et al., 2006), as also suggested in the case of SeNRs biosynthesized by *Rhodococcus aetherivorans* BCP1 (Presentato et al., 2018a). Conversely, upon pyruvate consumption, the biosynthesis of SeNPs was coupled with the appearance of intracellular accumulations (Figures 5 inlets **A1,D1**) resembling inclusions containing hydrophobic storage compounds. These observations are in line with the studies conducted on *Azospirillum brasilense* Sp245 and Sp7 strains, where heavy metal stress induced accumulation of polyester compounds (i.e., poly-3-hydroxybutyrate; Kamnev et al., 2002, 2005), which play a crucial role in bacterial tolerance toward environmental stresses, such as counteraction of a prolonged starvation phase (Olubai et al., 1998; Sun et al., 2000). Overall, these data reflected the way both SeITE02 and MPV1 strains responded to 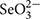 stress, suggesting a different behavior of these two environmental isolates once cultured under metabolically controlled conditions and as compared to their growth exploiting optimized ones.

From a physical–chemical point of view, a direct consequence of the high surface energy typical of material at the nanoscale (10^-9^ m) is the tendency of nanostructures to form aggregates to reach their thermodynamic stability (Goldstein et al., 1992). This represents a scenario that needs to be prevented to take advantages of the unique physical–chemical properties of nanomaterials (Roco, 1999). The stabilization of chemical nanomaterials is achieved by the addition of a variety of expensive and potentially toxic compounds able to generate electrostatic, steric or electrosteric repulsive interactions (Pachon and Rothenberg, 2008; Segets et al., 2011). Conversely, biogenic nanomaterials are featured by a natural thermodynamic stability, which mostly relies on their association with an organic material with a complex molecular composition that may act as electrosteric stabilizer (this topic has been extensively reviewed by Piacenza et al., 2018a). The stabilization of SeNPs biosynthesized by bacteria was earlier ascribed to their interaction with proteins and amphiphilic molecules, as reported in the case of *Rhodospirillum rubrum*, *Enterobacter cloacae* SLD1a-1, *Thauera selenatis* and *Rhodococcus aetherivorans* BCP1, to name a few (Kessi and Hanselmann, 2004; Yee et al., 2007; Butler et al., 2012; Presentato et al., 2018a). Thus, the organic material closely related to the biogenic SeNS detected (Figures 6, 7 and Supplementary Figure S2) likely contributed to their stabilization, as washing steps in hexane/water solution failed to completely remove it from the nanomaterial content, suggesting the existence of a sort of dynamic equilibrium between the organic and inorganic components constituting the biogenic extract (Presentato et al., 2016). In this context, the development of an electrosteric interaction between the organic material and the biogenic SeNS was supported by the detection of proteins and amphiphilic molecules through fluorescence spectroscopy (Figure 8 and Supplementary Figures S3, S4) and CLSM analyses (Figures 6B, 7B).

The uniqueness of nanomaterials relies on their enhanced physical–chemical properties as compared to their bulk counterpart, which makes nanostructures suitable for a variety of applications (Cao, 2004). For instance, photoelectric, photoconductive and semiconductive properties of Se at the nanoscale are emphasized as in the case of Se quantum dots, which, thanks to their high quantum confinement, are featured by high intrinsic fluorescence, whose emission wavelength red-shifts as the size decreases (Singh et al., 2010; Qian et al., 2017). Khalid et al. (2016) reported on the intrinsic photoluminescence properties of SeNPs (λ_em_ = 416 and 580 nm) upon excitation at 325 nm. Similarly, biogenic SeNS extracts and L-cys SeNPs showed the same behavior (λ_em_ = 416 nm) when excited at 380 nm, being also detected emission peaks at 450 and 480 nm (Figure 9A), which were not previously reported. The different excitation maximum peak (λ_exc_ = 380 nm) observed for biogenic SeNS extracts and L-cys SeNPs may depend on their bigger size (Table 1) as compared to those earlier synthesized (ca. 80 nm) by Khalid et al. (2016). Indeed, Shah et al. (2010) reported that the absorption maximum of SeNPs depended on the particle size, resulting centered in the region between 300 and 350 nm for particles up to 100 nm size, while a red-shift was observed as the SeNPs size increased. Nevertheless, the excitation spectra of biogenic SeNS extracts fixing the λ_em_ at either 530 or 640 nm revealed peaks in the 385–395 nm range (Supplementary Figures S6b,c), suggesting that the biogenic extracts contained few NPs of ca. 80 nm size. The polydispersity of the SeNS extracts may explain the PL dependency on the excitation wavelength, as by its variation the excitation of nanomaterials featured by a certain size can be achieved, therefore determining PL emission peaks centered at different wavelengths (Stengl and Henych, 2013). Further, Singh et al. (2010) indicated that the contribution to the PL emission of SeQDs upon excitation at 450 nm depended on both excitonic decay (λ_em_ = 520 nm) and surface defects (λ_em_ = 580 nm). Thus, the PL peak centered at 416 nm observed for SeNS (Figure 9A) was likely due to excitonic decay, which appears to be the highest contribute to PL emission. Indeed, the low degree of quantum confinement to which big SeNPs are subjected may result in less surface defects typically found in small NPs or QDs (Singh et al., 2010), therefore causing low PL emission above 500 nm. Moreover, all the biogenic SeNS extracts displayed an excitation wavelength-dependent PL emission (Figure 9, Supplementary Figure S5 and Supplementary Table S2), which agrees with the data reported by Qian et al. (2017). Finally, the main criteria to be satisfied for fluorescent biomarkers mostly relies on the possibility to excite them at a wavelength (λ_exc_ > 500 nm) that does not interfere with the intrinsic fluorescence of cellular components (e.g., collagens and flavins; λ_em_ 400–550 nm), therefore highlighting the need of probes able to emit fluorescence at wavelengths greater than 600 nm (Khalid et al., 2016). Indeed, the major drawbacks of fluorescent probes applied for clinical purposes is their ability to chemically react with the sample generating artifacts, as well as their toxicity (Jensen, 2012). In this context, since Se is an essential micronutrient, it is considered a preferential alternative to most of the chemical fluorescent compounds used for diagnostics (Khalid et al., 2016), as research to date showed also that Se-based nanomaterials resulted to be less toxic in comparison with other Se-compounds toward osteoblasts and human dermal fibroblasts (Tran et al., 2010; Ramos and Webster, 2012; Cremonini et al., 2016). Thus, the exploitation of the intrinsic fluorescence of SeNPs results advantageous, as they can be used as probe *per se* without the necessity of additional tags. In this regard, the excitation of biogenic SeNS extracts at 532 nm gave rise to PL emission at 640 nm, suggesting their promising suitability as potential photolumiscence tool for cell imaging, as well as therapeutic agents, considering the already established antimicrobial activity of SeNPs produced by SeITE02 strain against important nosocomial pathogen bacteria (Zonaro et al., 2015).

## Conclusion

The boosted physical–chemical properties and low intrinsic toxicity of selenium in the form of nanostructure, as well as the potentially amendable nanomaterial surface, empower the use of this chalcogen element by means of strategies aimed at generating new and non-toxic Se-based smart devices. With all this in mind, here, we showed that the bacterial strains *Stenotrophomonas maltophilia* SEITE02 and *Ochrobactrum* sp. MPV1 cultured under defined metabolic conditions revealed novel aspects in the respect of both 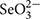 bioprocessing and the synthesis of different morphologies of Se-nanostructures. Particularly, this study contributed to expand the knowledge regarding the different behavior and strategies adopted by these microorganisms to counteract the toxicity of these oxyanions, while a fine characterization of the biogenic Se-nanostructure extracts highlighted their promising intrinsic photoluminescence property. Thus, the two different environmental isolates here investigated are worthy to be explored as suitable cell factories to biosynthesize valuable and tunable biogenic Se-nanomaterials, which represent an advanced technological and scientific frontier of scaled down material with multi-branched activities for biotechnological and biomedical applications.

## Author Contributions

EP and AP equally contributed to the scientific development of this study, namely: (i) experimental design, (ii) performing of the experiments, (iii) data interpretation, (iv) drafting the manuscript. EA performed scanning electron microscopy and Energy-dispersive X-ray spectroscopy. AS intellectually contributed to both the set up and the interpretation of spectrofluorimetric data. RT intellectually contributed to the development of the study and revised the whole manuscript. GV intellectually contributed interpreting the microbial physiology evidences derived from metal(loid) selective pressure exerted on the bacterial isolates investigated, as well as revising the manuscript. SL had a major intellectual and financial contribution during the development of the study, managing and directing the research as well as editing and revising the manuscript.

## Conflict of Interest Statement

The authors declare that the research was conducted in the absence of any commercial or financial relationships that could be construed as a potential conflict of interest.

## References

[B1] Ahmad M. S. Yasser M. M. Sholkamy E. N. Ali A. M. Mehanni M. M. (2015). Anticancer activity of biostabilized selenium nanorods synthesized by *Streptomyces bikiniensis* strain Ess_amA-1. *Int. J. Nanomed.* 10 3389–3401. 10.2147/IJN.S82707 26005349 PMC4428361

[B2] Alvarez H. M. Mayer F. Fabritius D. Steinbüchel A. (1996). Formation of intracytoplasmic lipid inclusions by *Rhodococcus opacus* strain PD630. *Arch. Microbiol.* 165 377–386. 10.1007/s002030050341 8661931

[B3] Ankamwar B. Chaudhary M. Sastry M. (2005). Gold nanoparticles biologically synthesized using tamarind leaf extract and potential application in vapour sensing. *Synth. React. Inorg. M.* 35 19–26. 10.1081/SIM-200047527

[B4] Appenzeller T. (1991). The man who dared to think small. *Science* 254:1300. 10.1126/science.254.5036.1300 17773595

[B5] Bartlett J. S. Voss K. J. Sathyendranath S. Vodacek A. (1998). Raman scattering by pure water and seawater. *Appl. Opt.* 37 3324–3332. 10.1364/AO.37.003324 18273291

[B6] Brus L. E. (1998). Chemical approaches to semiconductor nanocrystals. *J. Phys. Chem. Solids* 59 459–465. 10.1016/S0022-3697(97)00201-1

[B7] Butler C. S. Debieux C. M. Dridge E. J. Splatt P. Wright M. (2012). Biomineralization of selenium by selenate-respiring bacterium *Thauera selenatis*. *Biochem. Soc. Trans.* 40 1239–1243. 10.1042/BST20120087 23176461

[B8] Cao G. (2004). “Chapter 1, Introduction,” in *Nanostructures and Nanomaterials: Synthesis, Properties and Applications*, ed. Cao G. (London: Imperial College), 1–14. 10.1142/p305

[B9] Carmel-Harel O. Storz G. (2000). Roles of the glutathione- and thioredoxin dependent reduction systems in the *Escherichia coli* and *Saccharomyces cerevisiae* responses to oxidative stress. *Annu. Rev. Microbiol.* 54 439–461. 10.1146/annurev.micro.54.1.439 11018134

[B10] Chaudhary S. Umar A. Mehta S. K. (2016). Selenium nanomaterials: an overview of recent developments in synthesis, properties and potential applications. *Prog. Mater. Sci.* 83 270–329. 10.1016/j.pmatsci.2016.07.001

[B11] Cremonini E. Zonaro E. Donini M. Lampis S. Boaretti M. Dusi S. (2016). Biogenic selenium nanoparticles: characterization, antimicrobial activity and effects on human dendritic cells and fibroblasts. *Microb. Biotechnol.* 9 758–771. 10.1111/1751-7915.12374 27319803 PMC5072192

[B12] Di Gregorio S. Lampis S. Vallini G. (2005). Selenite precipitation by a rhizospheric strain of *Stenotrophomonas* sp. isolated from the root system of *Astragalus bisulcatus*: a biotechnological perspective. *Environ. Int.* 31 233–241. 10.1016/j.envint.2004.09.021 15661289

[B13] Eastoe J. Tabor R. F. (2014). “Chapter 6: Surfactants and nanoscience,” in *Colloidal Foundation of Nanoscience*, eds Berti D. Palazzo G. (London: Elsevier B.V.), 135–157. 10.1016/B978-0-444-59541-6.00006-0

[B14] El-Bayoumy K. Sinha R. (2004). Mechanisms of mammary cancer chemoprevention by organoselenium compounds. *Mutat. Res.* 551 181–197. 10.1016/j.mrfmmm.2004.02.023 15225592

[B15] Evans D. F. Wennerstrom H. (1994). “Chapter 8: Colloidal stability,” in *The Colloidal Domain: Where Physics, Chemistry, Biology and Technology Meet*, eds Evans D. F. Wennerstrom H. (New York, NY: Wiley-VCH).

[B16] Frassinetti S. Setti L. Corti A. Farrinelli P. Montevecchi P. Vallini G. (1998). Biodegradation of dibenzothiophene by a nodulating isolate of *Rhizobium meliloti*. *Can. J. Microbiol.* 44 289–297. 10.1139/w97-155 9606911

[B17] Goldstein A. N. Echer C. M. Alivisatos A. P. (1992). Melting in semiconductor nanocrystals. *Science* 256 1425–1427. 10.1126/science.256.5062.1425 17791609

[B18] Habashi F. (2013). “Selenium, physical and chemical properties,” in *Encyclopedia of Metalloproteins*, eds Kretsinger R. H. Uversky V. N. Permyakov E. A. (New York, NY: Springer Science+Business Media), 1924–1925. 10.1007/978-1-4614-1533-6_407

[B19] Haynes W. N. (2014). “Section 4, Properties of the elements and inorganic compounds,” in *Handbook of Chemistry and Physics*, 95th Edn, eds Haynes W. N. Lide D. R. Bruno T. J. (Boca Raton, FL: CRC Press/Taylor and Francis), 115–120.

[B20] Held K. D. Biaglow J. E. (1994). Mechanisms for the oxygen radical mediated toxicity of various thiol-containing compounds in cultured mammalian cells. *Radiat. Res.* 139 15–23. 10.2307/3578727 8016303

[B21] Holubova J. Cernosek Z. Cernoskova E. Cerna A. (2006). Crystallization of supercooled liquid of selenium: the comparison of kinetic analysis of both isothermal and non-isothermal DSC data. *Mater. Lett.* 60 2429–2432. 10.1016/j.matlet.2006.01.070

[B22] Huaglang R. P. (2002). *Handbook of Fluorescent Probes and Research Products*, 9th Edn. Eugene, OR: Molecular Probes.

[B23] Jensen E. C. (2012). Use of fluorescent probes: their effect on cell biology and limitations. *Anat. Rec.* 295 2031–2036. 10.1012/ar.22602 23060362

[B24] Jeong U. Camargo P. H. C. Lee Y. H. Xia Y. (2006). Chemical transformation: a powerful route to metal chalcogenide nanowires. *J. Mater. Chem.* 16 3893–3897. 10.1039/B606682H

[B25] Jixian Y. Wei W. Shanshan P. Fang M. Ang L. Dan W. (2015). Competitive adsorption of heavy metals by extracellular polymeric substances extracted from *Klebsiella* sp. J1. *Bioresour. Technol.* 196 533–539. 10.1016/j.biortech.2015.08.011 26291413

[B26] Kamnev A. A. Antonyuk L. P. Tugarova A. V. Tarantilis P. A. Polissiou M. G. Gardiner P. H. E. (2002). Fourier transform infrared spectroscopic characterization of heavy metal-induced metabolic changes in the plant-associated soil bacterium *Azospirillum brasilense* Sp7. *J. Mol. Struct.* 610 127–131. 10.1016/S0022-2860(02)00021-2

[B27] Kamnev A. A. Tugarova A. V. Antonyuk L. P. Tarantilis P. A. Polissiou M. G. Gardiner P. H. E. (2005). Effects of heavy metals on plant-associated rhizobacteria: comparison of endophytic and non-endophytic strains of *Azospirillum brasilense*. *J. Trace Elem. Med. Biol.* 19 91–95. 10.1016/j.jtemb.2005.03.002 16240678

[B28] Kessi J. Hanselmann K. W. (2004). Similarities between the abiotic reduction of selenite with glutathione and the dissimilatory reaction mediated by *Rhodospirillum rubrum* and *Escherichia coli*. *J. Biol. Chem.* 279 50662–50669. 10.1074/jbc.M405887200 15371444

[B29] Kessi J. Ramuz M. Wehrli E. Spycher M. Bachofen R. (1999). Reduction of selenite and detoxification of elemental selenium by the phototrophic bacterium *Rhodospirillum rubrum*. *Appl. Environ. Microbiol.* 65 4734–4740. 10543779 10.1128/aem.65.11.4734-4740.1999 PMC91637

[B30] Khalid A. Tran P. A. Norello R. Simpson D. A. O’Connor A. J. Tomljenovic-Hanic S. (2016). Intrinsic fluorescence of selenium nanoparticles for cellular imaging applications. *Nanoscale* 8 3376–3385. 10.1038/c5nr08771f 26792107

[B31] Kulp A. Kuehn M. J. (2010). Biological functions and biogenesis od secreted bacterial outer membrane vesicles. *Annu. Rev. Microbiol.* 64 163–184. 10.1146/annurev.micro.091208.073413 20825345 PMC3525469

[B32] Kumar C. Sujhitha P. Mamidyala S. Usharani P. Das B. Reddy C. (2014). Ochrosin, a new biosurfactant produced by halophilic *Ochrobactrum* sp. strain BS-206 (MTCC 5720): purification, characterization and its biological evaluation. *Process Biochem.* 49 1708–1717. 10.1016/j.procbio.2014.07.004

[B33] Lakowicz J. R. (1999). “Chapter 16: Protein fluorescence,” in *Principles of Fluorescence Spectroscopy*, ed. Lakowicz J. R. (New York, NY: Springer Science+Business Media), 446–485.

[B34] Lampis S. Santi C. Ciurli A. Andreolli M. Vallini G. (2015). Promotion of arsenic phytoextraction efficacy in the fern *Pteris vittata* by the inoculation of As-resistant bacteria: a soil bioremediation perspective. *Front. Plant Sci.* 6:80. 10.3389/fpls.2015.00080 25741356 PMC4332284

[B35] Lampis S. Zonaro E. Bertolini C. Cecconi D. Monti F. Micaroni M. (2017). Selenite biotransformation and detoxification by *Stenotrophomonas* maltophilia SeITE02: novel clues on the route to bacterial biogenesis of selenium nanoparticles. *J. Hazard. Mater.* 324 3–14. 10.1016/j.hazmat.2016.02.035 26952084

[B36] Li Q. Chen T. Yang F. Liu J. Zheng W. (2010). Facile and controllable one-step fabrication of selenium nanoparticles assisted by L-cysteine. *Mater. Lett.* 64 614–617. 10.1016/j.matlet.2009.12.019

[B37] Li X. Xu H. Chen Z. S. Chen G. (2011). Biosynthesis of nanoparticles by microorganisms and their applications. *J. Nanomater.* 2011:270974 10.1155/2011/270974

[B38] Losi M. E. Frankenberger W. T. (1997). Reduction of selenium oxyanions by *Enterobacter cloacae* SLD1a-1: isolation and growth of the bacterium and its expulsion of selenium particles. *Appl. Environ. Microbiol.* 63 3079–3084. 16535668 10.1128/aem.63.8.3079-3084.1997 PMC1389223

[B39] McBroom A. J. Kuehn M. J. (2007). Release of outer membrane vesicles by gram-negative bacteria is a novel envelope stress response. *Mol. Microbiol.* 63 545–558. 10.1111/j.1365-2958.2006.05522.x 17163978 PMC1868505

[B40] Mehdi Y. Hornick J. L. Istasse L. Dufrasne I. (2013). Selenium in the environment, metabolism and involvement in body functions. *Molecules* 18 3292–3311. 10.3390/molecules18033292 23486107 PMC6270138

[B41] Olubai O. R. Caudales A. Atkinson A. Neyra C. A. (1998). Differences in chemical composition between non-flocculated and flocculated *Azospirillum brasilense* cd. *Can. J. Microbiol.* 44 386–390. 10.1139/w98-002

[B42] Oves M. Khan M. S. Zaidi A. Ahmed A. S. Ahmed F. Ahmad E. (2013). Antibacterial and cytotoxic efficacy of extracellular silver nanoparticles biofabricated from chromium reducing novel OS4 strain of *Stenotrophomonas* maltophilia. *PLoS One* 8:e59140. 10.137/journal.pone.0059140 23555625 PMC3605433

[B43] Pachon L. D. Rothenberg G. (2008). Transition-metal nanoparticles: synthesis, stability and the leaching issue. *Appl. Organomet. Chem.* 22 288–299. 10.1002/aoc.1382

[B44] Patton T. G. Rice K. C. Foster M. K. Bayles K. W. (2005). The *Staphylococcus aureus* cidC gene encodes a pyruvate oxidase that affects acetate metabolism and cell death in stationary phase. *Mol. Microbiol.* 56 1664–1674. 10.1111/j.1365-2958.2005.04653.x 15916614

[B45] Pereira A. G. Gerolis L. G. L. Goncalves L. S. Pedrosa T. A. Neves M. J. (2018). Selenized *Saccharomyces cerevisiae* cells are a green dispenser of nanoparticles. *Biomed. Phys. Eng. Express* 4:035028 10.1088/2057-1976/aab524

[B46] Piacenza E. Presentato A. Turner R. J. (2018a). Stability of biogenic metal(loid) nanomaterials related to the colloidal stabilization theory of chemical nanostructures. *Crit. Rev. Biotechnol.* 38 1137–1156. 10.1080/07388551.2018.1440425 29480081

[B47] Piacenza E. Presentato A. Zonaro E. Lampis S. Vallini G. Turner R. J. (2018b). Selenium and tellurium nanomaterials. *Phys. Sci. Rev.* 3:16 10.1515/psr-2017-0100

[B48] Piacenza E. Presentato A. Zonaro E. Lemire J. A. Demeter M. Vallini G. (2017). Antimicrobial activity of biogenically produced spherical Se-nanomaterials embedded in organic material against *Pseudomonas aeruginosa* and Staphylococcus aureus strains on hydroxyapatite-coated surfaces. *Microb. Biotechnol.* 10 804–818. 10.1111/1751-7915.12700 28233476 PMC5481514

[B49] Presentato A. Piacenza E. Anikovkiy M. Cappelletti M. Zannoni D. Turner R. J. (2016). *Rhodococcus aetherivorans* BCP1 as cell factory for the production of intracellular tellurium nanorods under aerobic conditions. *Microb. Cell Fact.* 15:204. 10.1186/12934-016-0602-8 27978836 PMC5157098

[B50] Presentato A. Piacenza E. Anikovkiy M. Cappelletti M. Zannoni D. Turner R. J. (2018a). Biosynthesis of selenium-nanoparticles and -nanorods as a product of selenite bioconversion by the aerobic bacterium *Rhodococcus aetherivorans* BCP1. *N. Biotechnol.* 41 1–8. 10.1016/j.nbt.2017.11.002 29174512

[B51] Presentato A. Piacenza E. Darbandi A. Anikovskiy M. Cappelletti M. Zannoni D. (2018b). Assembly, growth and conductive properties of tellurium nanorods produced by *Rhodococcus aetherivorans* BCP1. *Sci. Rep.* 8:3923. 10.1038/s41598-018-22320-x 29500440 PMC5834534

[B52] Qian F. Li X. Tang L. Lai S. K. Lu C. Lau S. P. (2017). Selenium quantum dots: preparation, structure and properties. *Appl. Phys. Lett.* 110:053104 10.1063/1.4975358

[B53] Ramos J. F. Webster T. J. (2012). Cytotoxicity of selenium nanoparticles in rat dermal fibroblasts. *Int. J. Nanomed.* 7 3907–3914. 10.2147/IJN.S33767 22915842 PMC3418168

[B54] Reyes L. H. Marchante-Gayón J. M. García Alonso J. I. Sanz-Medel A. (2006). Application of isotope dilution analysis for the evaluation of extraction conditions in the determination of total selenium and selenomethionine in yeast-based nutritional supplements. *J. Agric. Food Chem.* 54 1557–1563. 10.1021/jf0523768 16506800

[B55] Rice K. C. Bayles K. W. (2008). Molecular control of bacterial death and lysis. *Microbiol. Mol. Biol. Rev.* 72 85–109. 10.1128/MMBR.00030-07 18322035 PMC2268280

[B56] Rice K. C. Nelson J. B. Patton T. G. Yang S. J. Bayles K. W. (2005). Acetic acid induces expression of the *Staphylococcus aureus* cidABC and lrgAB murein hydrolase regulator operons. *J. Bacteriol.* 187 813–821. 10.1128/JB.187.3.813-821.2005 15659658 PMC545714

[B57] Roco M. C. (1999). Nanoparticles and nanotechnology research. *J. Nanopart. Res.* 1 1–6. 10.1023/A:1010093308079

[B58] Segets D. Marczak R. Schaufer S. Paula C. Gnichwitz J. F. Hirsch A. (2011). Experimental and theoretical studies of the colloidal stability of nanoparticles: a general interpretation based on stability maps. *ACS Nano* 5 4658–4669. 10.1021/nn200465b 21545143

[B59] Shah C. P. Dwivedi C. Singh K. K. Kumar M. Bajaj P. N. (2010). Riley oxidation: a forgotten name reaction for synthesis of selenium nanoparticles. *Mater. Res. Bull.* 45 1213–1217. 10.1016/j.materresbull.2010.05.013

[B60] Shirsat S. Kadam A. Naushad M. Mane R. S. (2015). Selenium nanostructures: microbial synthesis and applications. *RSC Adv.* 5 92799–92811. 10.1039/C5RA17921A

[B61] Singh S. C. Mishra S. K. Srivastava R. K. Gopal R. (2010). Optical properties of selenium quantum dots produced with laser irradiation of water suspended Se nanoparticles. *J. Phys. Chem. C* 114 17374–17384. 10.1021/jp105037w

[B62] Somerville G. A. Said-Salim B. Wickman J. M. Raffel S. J. Kreiswirth B. N. Musser J. M. (2003). Correlation of acetate catabolism and growth yield in *Staphylococcus aureus*: implications for host-pathogen interactions. *Infect. Immun.* 71 4724–4732. 10.128/IAI.71.8.4724-4732.2003 12874354 PMC166023

[B63] Srivastava N. Mukhopadhyay M. (2015). Green synthesis and structural characterization of selenium nanoparticles and assessment of their antimicrobial property. *Bioprocess Biosyst. Eng.* 38 1723–1730. 10.1007/s00449-015-1413-8 25972036

[B64] Stengl V. Henych J. (2013). Strongly luminescent monolayered MoS2 prepared by effective ultrasound exfoliation. *Nanoscale* 5 3387–3394. 10.1039/C3NR00192J 23467444

[B65] Sun J. Peng X. Van Impe J. Vanderleyden J. (2000). The ntrB and ntrC genes are involved in the regulation of poly-3-hydroxybutyrate biosynthesis by ammonia in *Azospirillum brasilense* Sp7. *Appl. Environ. Microbiol.* 66 113–117. 10.1128/AEM.66.1.113-117.2000 10618211 PMC91793

[B66] Tinggi U. (2008). Selenium: its role as antioxidant in human health. *Environ. Health Prev. Med.* 13 102–108. 10.1007/s12199-007-0019-4 19568888 PMC2698273

[B67] Tomei F. A. Barton L. L. Lemanski C. L. Zocco T. G. Fink N. H. Sillerud L. O. (1995). Transformation of selenate and selenite to elemental selenium by *Desulfovibrio desulfuricans*. *J. Ind. Microbiol.* 14 329–336. 10.1007/BF01569947

[B68] Tran P. A. Sarin L. Hurt R. H. Webster T. J. (2010). Differential effects of nanoselenium doping on health and cancerous osteoblasts in coculture on titanium. *Int. J. Nanomed.* 5 351–358. 10.2147/IJN.S7289 PMC2875729 20517480

[B69] Wadhwani S. A. Shedbalkar U. U. Singh R. Chopade B. A. (2016). Biogenic selenium nanoparticles: current status and future prospects. *Appl. Microbiol. Biotechnol.* 100 2555–2566. 10.1007/s00253-016-7300-7 26801915

[B70] Wang T. Yang L. Zhang B. Liu J. (2010). Extracellular biosynthesis and transformation of selenium nanoparticles and application in H2O2 biosensor. *Colloid Surf. B Biointerfaces* 80 94–102. 10.10167/j.colsurfb.2010.05.041 20566271

[B71] Yee N. Ma J. Dalia A. Boonfueng T. Kobayashi D. Y. (2007). Se(VI) reduction and the precipitation of Se(0) by the facultative bacterium *Enterobacter cloacae* SLD1a-1 are regulated by FNR. *Appl. Environ. Microbiol.* 73 1914–1920. 10.1128/AEM.02542-06 17261520 PMC1828800

[B72] Yefimova S. L. Gural’chuk G. Y. Sorokin A. V. Malyukin Y. V. Borovoy I. A. Lubyanaya A. S. (2008). Hydrophobicity effect of interaction between organic molecules in nanocages of surfactant micelle. *J. Appl. Spectros.* 75 658–663. 10.1007/s10812-008-9108-4

[B73] Yuwen L. Wang L. (2013). “Chapter 11.5, Nanoparticles and quantum dots,” in *Handbook of Chalcogen Chemistry: New Perspectives in Sulfur, Selenium and Tellurium*, 2nd Edn, eds Devillanova F. Mont W. W. Du (Cambridge: The Royal Society of Chemistry), 232–260.

[B74] Zannoni D. Borsetti F. Harrison J. J. Turner R. J. (2008). The bacterial response to the chalcogen metalloids Se and Te. *Adv. Microb. Physiol.* 53 1–71. 10.1016/S0065-2911(07)53001-8 17707143

[B75] Zhang B. Ye X. Dai W. Hou W. Zuo F. Xie Y. (2006). Biomolecule-assisted synthesis of single-crystalline selenium nanowires and nanoribbons via a novel flake-cracking mechanism. *Nanotechnology* 17 385–390. 10.1088/0957-4484/17/2/007

[B76] Zhang L. Li D. Gao P. (2012). Expulsion of selenium/protein nanoparticles through vesicle-like structures by *Saccharomyces cerevisiae* under microaerophilic environment. *World J. Microbiol. Biotechnol.* 28 3381–3386. 10.1007/s11274-012-1150-y 22956051

[B77] Zhang W. Chen Z. Liu H. Zhang L. Gao P. Li D. (2011). Biosynthesis and structural characteristics of selenium nanoparticles by *Pseudomonas alcaliphila*. *Colloid Surf. B Biointerfaces* 88 196–201. 10.1016/j.colsurfb.2011.06.031 21752611

[B78] Zheng-Bo Y. Qing L. Chuan-chuan L. Tian-hu C. Jin W. (2015). Component analysis and heavy metal adsorption ability of extracellular polymeric substances (EPS) from sulfate reducing bacteria. *Bioresour. Technol.* 194 399–402. 10.1016/j.biortech.2015.07.042 26210529

[B79] Zonaro E. Lampis S. Turner R. J. Qazi S. J. S. Vallini G. (2015). Biogenic selenium and tellurium nanoparticles synthesized by environmental microbial isolates efficaciously inhibit bacterial planktonic cultures and biofilms. *Front. Microbiol.* 6:584. 10.3389/fmicb.2015.00584 26136728 PMC4468835

[B80] Zonaro E. Piacenza E. Presentato A. Monti F. Dell’Anna R. Lampis S. (2017). Ochrobactrum sp. MPV1 from a dump of roasted pyrites can be exploited as bacterial catalyst for the biogenesis of selenium and tellurium nanoparticles. *Microb. Cell Fact.* 16:215. 10.1186/212934-0826-2 29183326 PMC5704588

