# Supplementary material for: Physical–Chemical Properties of Biogenic Selenium Nanostructures Produced by Stenotrophomonas maltophilia SeITE02 and Ochrobactrum sp. MPV1
Source: Front Microbiol. 2018 Dec 19;9:3178. doi: 10.3389/fmicb.2018.03178 (PMC6306038; doi:10.3389/fmicb.2018.03178)
Supplement: Supplementary file 1 [file Table_1.docx]

Supporting information for:

**Physical-chemical properties of biogenic selenium nanostructures produced by *Stenotrophomonas maltophilia* SeITE02 and *Ochrobactrum sp.* MPV1**

Elena Piacenza^1,2,*^, Alessandro Presentato^1,*,†^, Emmanuele Ambrosi^3^, Adolfo Speghini^4^, Raymond J. Turner^2^, Giovanni Vallini^1,†^ and Silvia Lampis^1^

^1^Environmental Microbiology and Microbial Biotechnology Laboratory, Department of Biotechnology, University of Verona, Verona, Italy

^2^Department of Biological Sciences, University of Calgary, Calgary, AB, Canada

^3^Department of Molecular Sciences and Nanosystems, Ca’Foscari University, Venezia, Italy

^4^Nanomaterials Research Group, Department of Biotechnology, University of Verona and INSTM, RU Verona, Verona, Italy

^*^These authors contributed equally to the work.

^†^Corresponding author: giovanni.vallini@univr.it; alessandro.presentato@univr.it


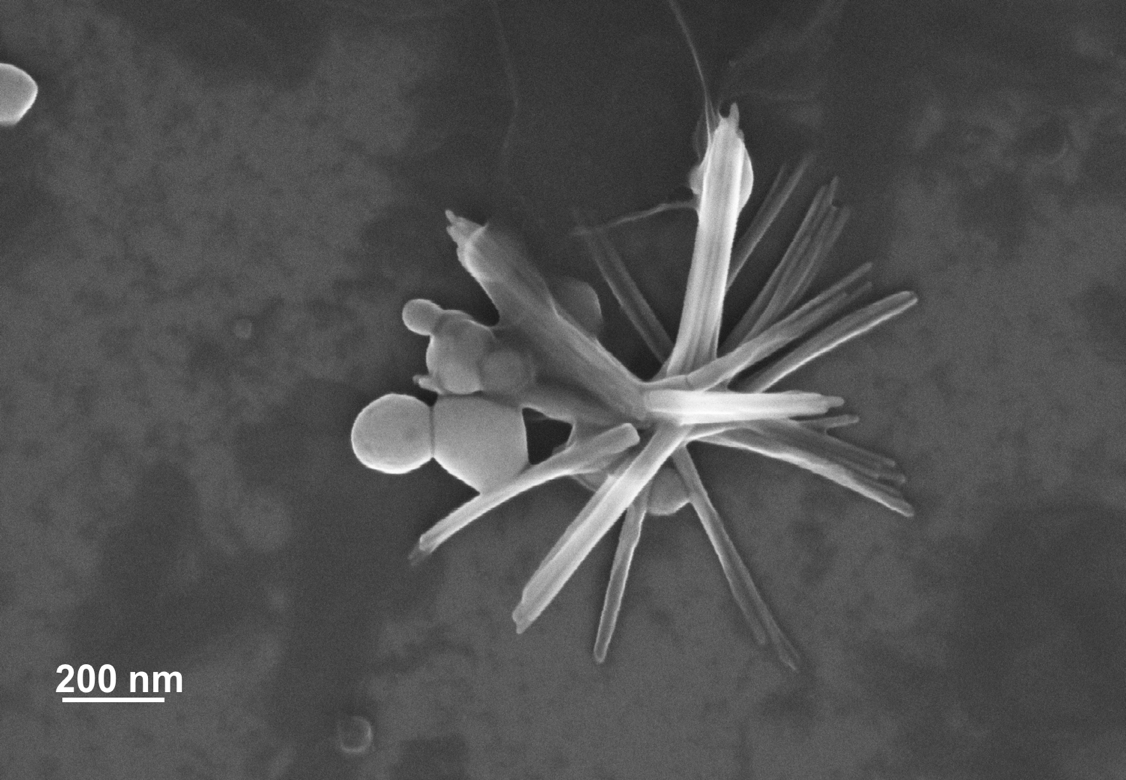


**Figure S1:** Scanning electron micrograph of SeNS_MPV1-G_e_ showing SeNPs and SeNRs.


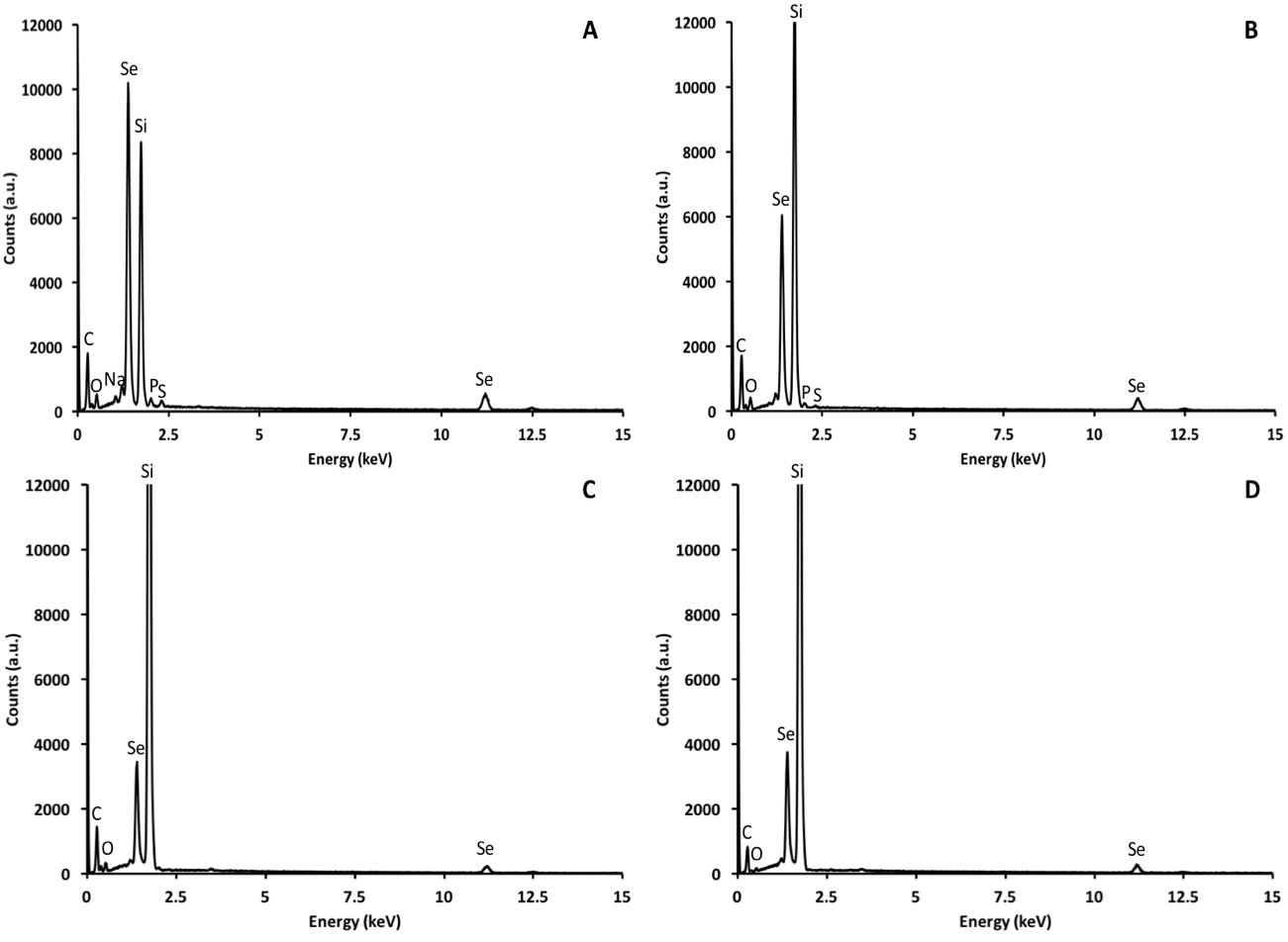


**Figure S2:** EDX spectra of SeNPs_SeITE02-G_e_ (a), SeNPs_SeITE02-P_e_ (b), SeNS_MPV1-G_e_ (c), and SeNS_MPV1-P_e_ (d).

**Table S1:** growth conditions adopted to culture the environmental bacterial isolates to generate SeNS extracts and their acronym.

| **Bacterial strain** | **Growth conditions of production** | **Acronym of SeNS extracts** |
| --- | --- | --- |
| *Stenotrophomonas maltophilia* SeITE02 | DM ^a^ medium amended with glucose (0.5% v/v) and SeO_3_^2-^ (0.5 mM) | SeNPs_SeITE02-G_e_ |
|  | DM ^a^ medium amended with pyruvate (0.5% v/v) and SeO_3_^2-^ (0.5 mM) | SeNPs_SeITE02-P_e_ |
| *Ochrobactrum* sp. MPV1 | DM ^a^ medium amended with glucose (0.5% v/v) and SeO_3_^2-^ (0.5 mM) | SeNS_MPV1-G_e_ |
|  | DM ^a^ medium amended with pyruvate (0.5% v/v) and SeO_3_^2-^ (0.5 mM) | SeNPs_MPV1-P_e_ |

^a^ Defined Medium (Frassinetti et al., 1998)


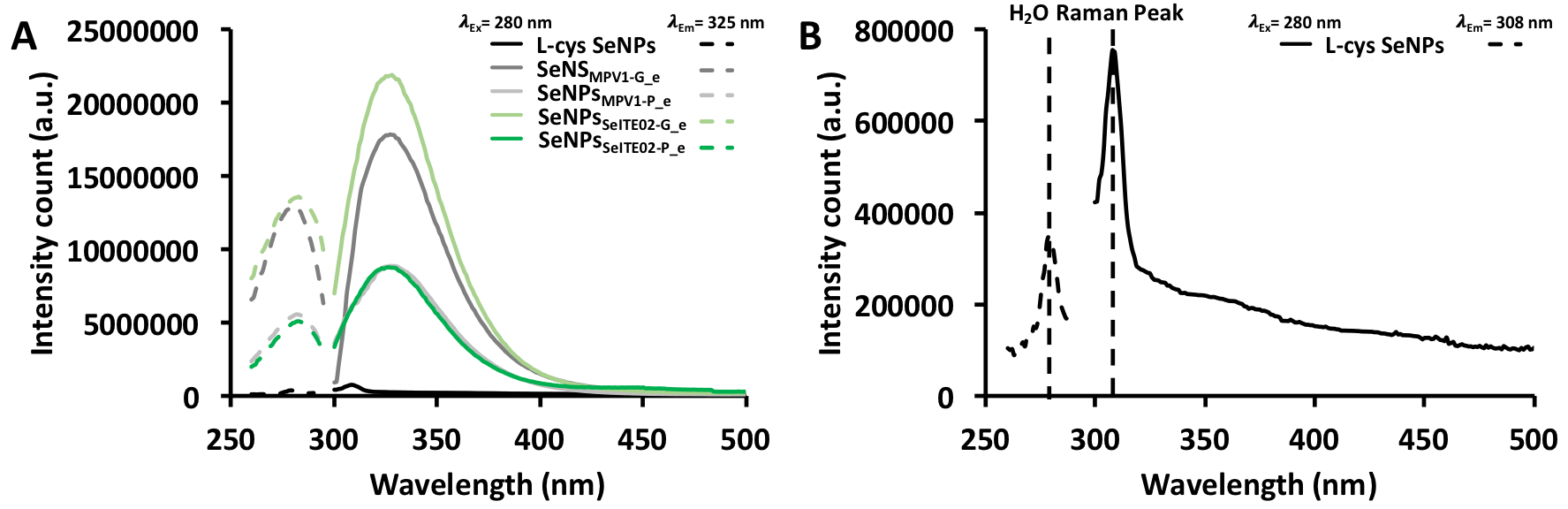


**Figure S3:** Fluorescence emission and excitation spectra of unlabeled biogenic SeNS extracts and chemically synthesized L-cys SeNPs (a), while, for clarity, in (b) is represented resolution of L-cys SeNPs spectra. The dashed black line indicates the water Raman peak.

**
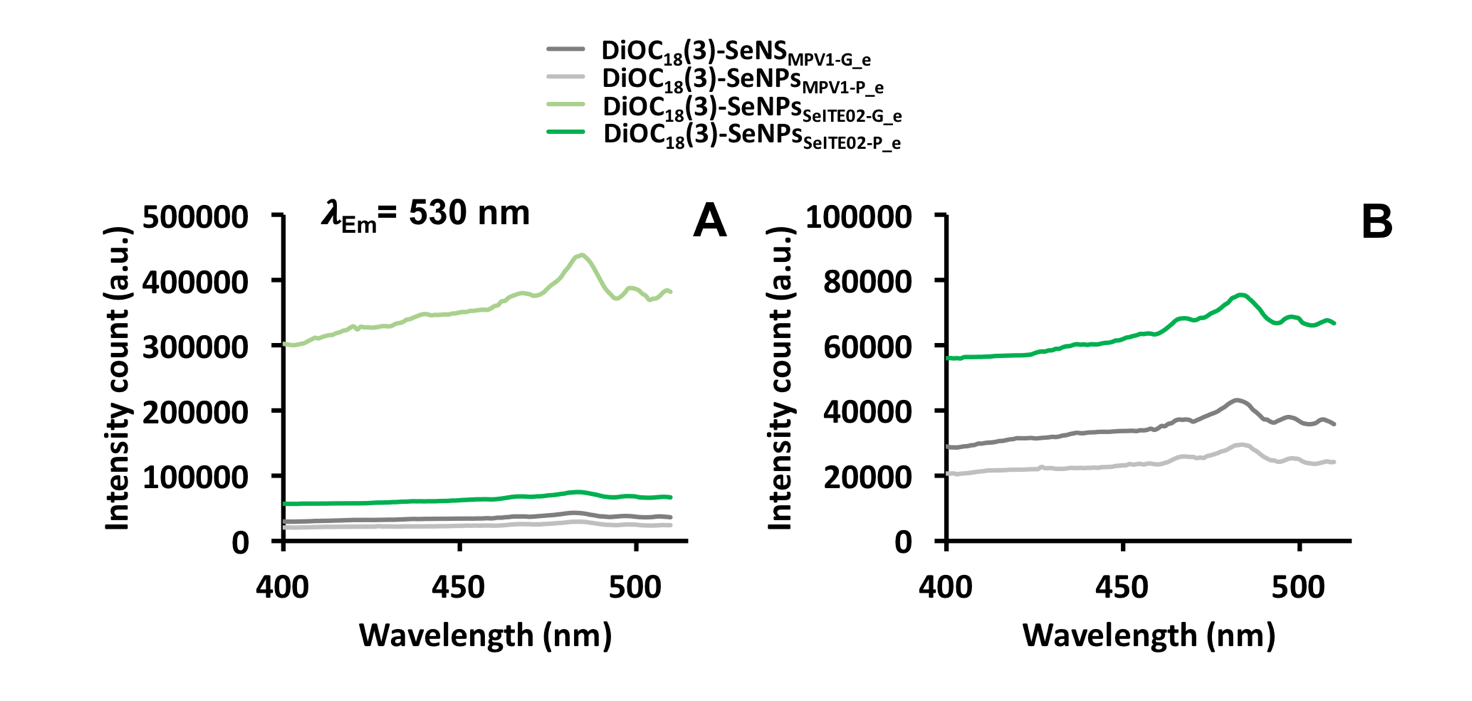
**

**Figure S4:** Fluorescence excitation spectra of the biogenic SeNS extracts labelled with the lipophilic tracer DiOC_18_(3) (a), while, for clarity, in (b) is represented a better resolution of those samples with low fluorescent signal.

**Table S2**: SeNS photoluminescence’s dependency on the excitation wavelength.

| **Emission wavelength (nm)** | | | | | |
| --- | --- | --- | --- | --- | --- |
| **λ_exc_ (nm)** | **L-cys SeNPs** | **SeNPs_SeITE02-G_e_** | **SeNPs_SeITE02-P_e_** | **SeNS_MPV1-G_e_** | **SeNPs_MPV1-P_e_** |
| 380 | 428 | 415 | 416 | 416 | 415 |
| 400 | 439 | 437 | 438 | 438 | 438 |
| 420 | 460 | 459 | 459 | 460 | 459 |
| 450 | 507 | 507 | 506 | 505 | 505 |
| 485 | 529 | 530 | 531 | 530 | 530 |
| 510 | 636 | 638 | 636 | 638 | 637 |
| 532 | 640 | 641 | 640 | 641 | 640 |
| 566 | 670 | 672 | 669 | 671 | 670 |
| 589 | 680 | 680 | 678 | 682 | 681 |
| **R^2^** | 0.96 | 0.96 | 0.96 | 0.96 | 0.96 |


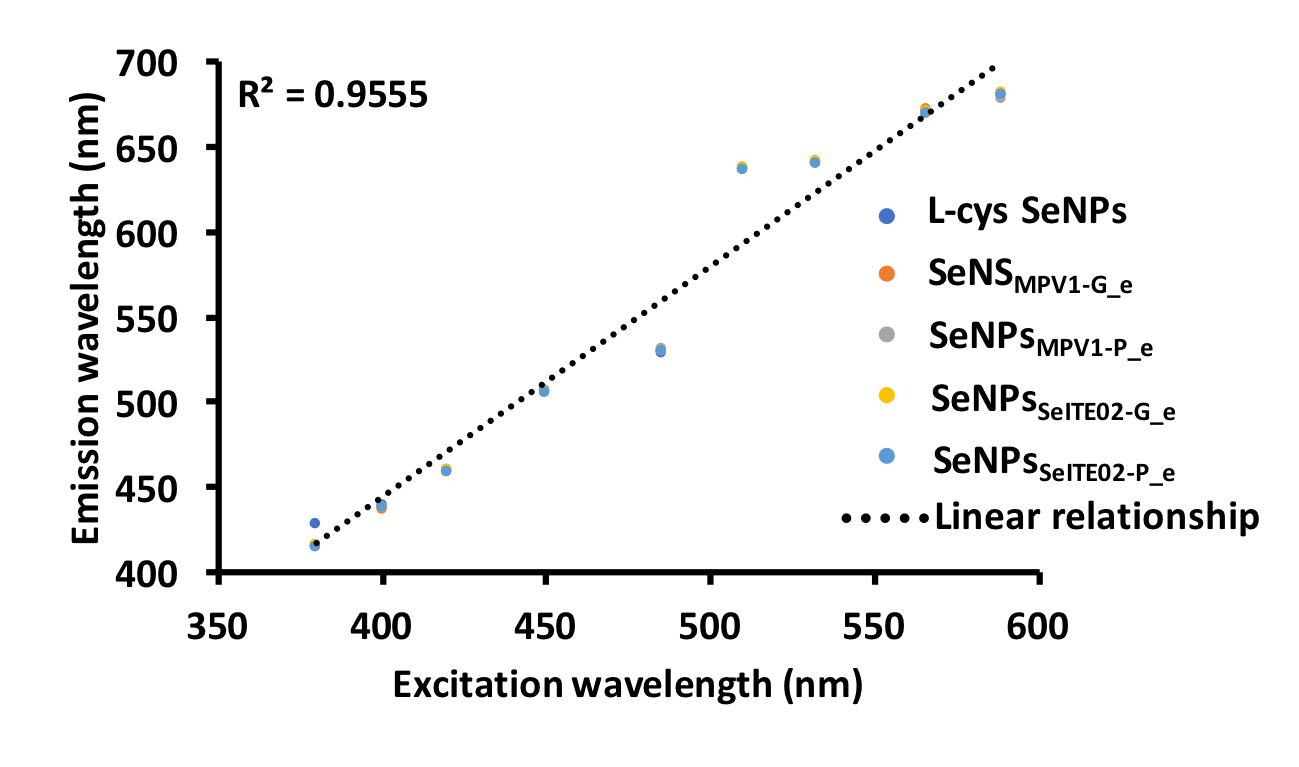


**Figure S5:** Linear relationship between the photoluminescence emission wavelength and the excitation wavelengths.

**
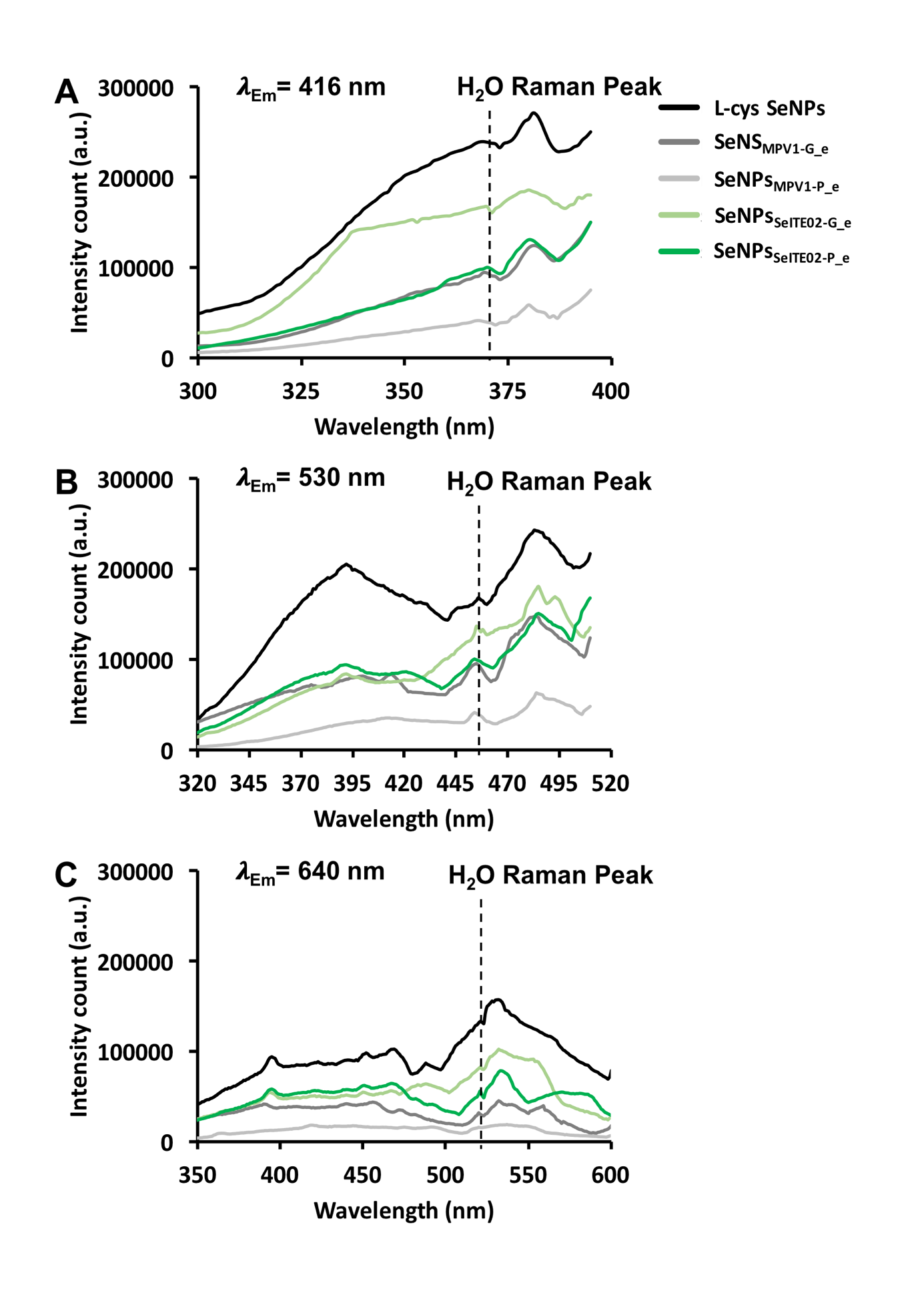
**

**Figure S6:** Photoluminescence excitation spectra of the unlabeled chemically and biogenically synthesized SeNS, setting the emission wavelength at 416 nm (a), 530 nm (b) and 640 nm (c). The dashed black line indicates the water Raman peak.

**Table S3**: SeNS photoluminescence excitation dependency on the emission wavelength.

| **Excitation wavelength (nm)** | | | | | |
| --- | --- | --- | --- | --- | --- |
| **λ_em_ (nm)** | **L-cys SeNPs** | **SeNPs_SeITE02-G_e_** | **SeNPs_SeITE02-P_e_** | **SeNS_MPV1-G_e_** | **SeNPs_MPV1-P_e_** |
| 416 | 381 | 380 | 380 | 381 | 380 |
| 440 | 402 | 401 | 400 | 401 | 400 |
| 460 | 421 | 419 | 418 | 420 | 420 |
| 490 | 451 | 448 | 449 | 449 | 450 |
| 510 | 466 | 466 | 466 | 465 | 464 |
| 530 | 483 | 485 | 485 | 484 | 484 |
| 550 | 503 | 506 | 505 | 505 | 502 |
| 570 | 509 | 512 | 510 | 511 | 510 |
| 640 | 533 | 532 | 533 | 532 | 533 |
| **R^2^** | 0.94 | 0.94 | 0.95 | 0.95 | 0.95 |


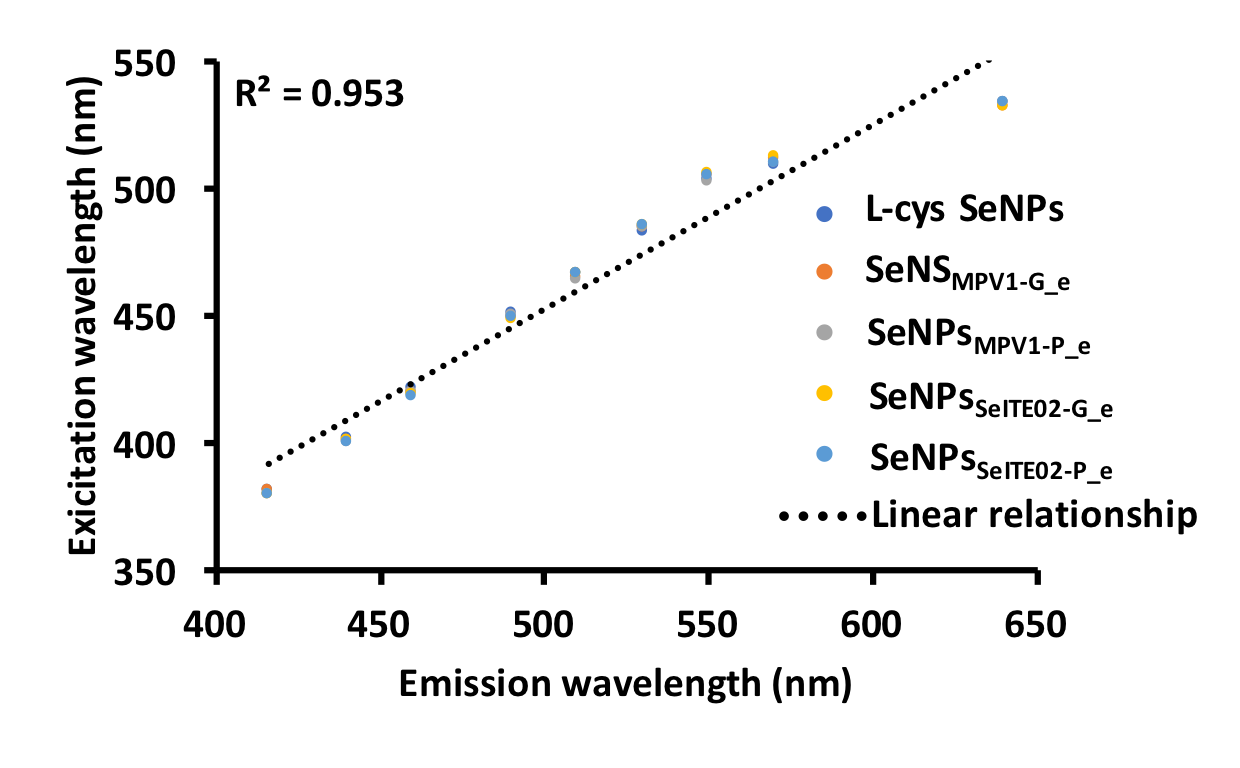


**Figure S7:** Linear relationship between the photoluminescence excitation wavelength and the emission wavelengths.
